# Constructing metagenome-assembled genomes for almost all components in a real bacterial consortium for binning benchmarking

**DOI:** 10.1186/s12864-022-08967-x

**Published:** 2022-11-10

**Authors:** Ziyao Wu, Yuxiao Wang, Jiaqi Zeng, Yizhuang Zhou

**Affiliations:** 1 Guangxi Key Laboratory of Environmental Exposomics and Entire Lifecycle Health, School of Public Health, Guilin Medical University, Guilin, 541199 Guangxi China; 2 Insitute of Pathogeny Biology, School of Basic Medicine, Guilin Medical University, Guilin, 541199 Guangxi China

**Keywords:** Binning, Metagenomics, Composition, Abundance, Benchmark

## Abstract

**Background:**

So far, a lot of binning approaches have been intensively developed for untangling metagenome-assembled genomes (MAGs) and evaluated by two main strategies. The strategy by comparison to known genomes prevails over the other strategy by using single-copy genes. However, there is still no dataset with all known genomes for a real (not simulated) bacterial consortium yet.

**Results:**

Here, we continue investigating the real bacterial consortium F1RT enriched and sequenced by us previously, considering the high possibility to unearth all MAGs, due to its low complexity. The improved F1RT metagenome reassembled by metaSPAdes here utilizes about 98.62% of reads, and a series of analyses for the remaining reads suggests that the possibility of containing other low-abundance organisms in F1RT is greatly low, demonstrating that almost all MAGs are successfully assembled. Then, 4 isolates are obtained and individually sequenced. Based on the 4 isolate genomes and the entire metagenome, an elaborate pipeline is then in-house developed to construct all F1RT MAGs. A series of assessments extensively prove the high reliability of the herein reconstruction. Next, our findings further show that this dataset harbors several properties challenging for binning and thus is suitable to compare advanced binning tools available now or benchmark novel binners. Using this dataset, 8 advanced binning algorithms are assessed, giving useful insights for developing novel approaches. In addition, compared with our previous study, two novel MAGs termed FC8 and FC9 are discovered here, and 7 MAGs are solidly unearthed for species without any available genomes.

**Conclusion:**

To our knowledge, it is the first time to construct a dataset with almost all known MAGs for a not simulated consortium. We hope that this dataset will be used as a routine toolkit to complement mock datasets for evaluating binning methods to further facilitate binning and metagenomic studies in the future.

**Supplementary Information:**

The online version contains supplementary material available at 10.1186/s12864-022-08967-x.

## Background

Thanks to technological advancements, the shift from gene-centric [1, 2] to genome-centric studies [3–7] was possible, and this has facilitated deepening the understanding of the functional capacity, evolution trend, ecological role, and composition structure of the microbial communities. However, assembling individual genomes directly from metagenomes is extremely challenging, possibly due to (but not limited to) the coexistence of intragenomic or intergenomic repeats. To address this problem, binning, the process to aggregate unassembled reads or assemblies into individual or population genomes, has been proposed and exemplified by several documented studies [3–9]. Meanwhile, an increasing number of binning tools have been intensively developed [10].

For assessing binning performance regarding completeness and purity, two types of datasets are generally used. One is a mock dataset with all known genomes, which can be established through *in silico* simulation of sequencing reads [11–14], *in silico* combination of randomly-selected reads sequenced from isolate genomes [15], or ab initio sequencing of synthetic consortiums comprising genome-sequenced strains [16, 17]. The other is a dataset sequenced from a real (not simulated) consortium with a negligible fraction or even no known genomes. Correspondingly, there are two main strategies for evaluating binning performance, the former relying on reference to known genomes and the latter with reliance on the conserved or lineage-specific essential single-copy genes (SCGs), such as CheckM [18]. In general, the latter is less than ideal, compared with the former, due to both the uneven distribution of SCGs across a genome and their low number, typically accounting for < 10% of all genes [19]. Accordingly, the former is preferred for mock datasets, while the latter is indispensably used for the dataset of real consortiums. For example, Alneberg et al. assessed binning performance by using the former for mock datasets and the latter for real datasets [20]. If the dataset for a real consortium has all known genomes, the former can be leveraged to achieve a complete and accurate assessment of binning tools. However, to our knowledge, no datasets with all known genomes for all components in not simulated consortiums have been established so far.

F1RT, a real (not simulated) bacterial consortium enriched in the laboratory and in-depth described previously by us, is dramatically simple [only 7 components (species) found in the previous study including both high- and low-abundance ones] [21], providing an opportunity to uncover all its metagenome-assemble genomes (MAGs) to construct the first dataset with all known genomes for a real consortium. If so, F1RT can function as a thoroughly independent, and in-depth benchmark of binning tools. In this context, F1RT becomes the focal in this study.

Here, we isolated 4 components from F1RT and found that these isolates, fortunately, separated the remaining except for FC8-9 based on sequencing coverage. Therefore, an elaborate in-house pipeline was developed to obtain almost all its MAGs. A series of assessments further confirmed that these MAGs are outstandingly reliable. Subsequently, we found that F1RT is suitable to function as a benchmark, due to several properties suitable for assessing the mainstream binning tools available. Finally, as a test, we used these MAGs to benchmark 8 advanced stand-alone binning tools and found that almost all of them poorly bin a simple metagenome even like F1RT, indicating that there is room to improve binning. We envisage that this dataset will become a useful benchmark in complementing mock datasets for evaluating binning methods and further facilitate binning and metagenomic studies in the future.

## Results

### Reassembling the F1RT metagenome for almost all components

The herein research object F1RT had been deeply sequenced by Illumina technology with a size of 12.8 Gb raw metagenomic reads. Such deep sequencing yielded a broad range of sequencing depth from ~ 1015× for FC1 to ~ 16× for FC7 (see Table 1 in [21]). As previous studies showed that metaSPAdes is the best among the well-known assemblers [22–24], metaSPAdes was utilized to *de novo* reassemble the F1RT metagenome. A total of 31.65 Mb was *de novo* reassembled into 3,852 scaffolds (≥ 500 bp) by using the assembler metaSPAdes (version v3.15.4; default parameters) [25], accounting for about 98.62% of reads, which was calculated *via* mapping against all edge connections in the output file “assembly_graph.fastg” of metaSPAdes. Further tracking found that all edges are included in the final 3,852 scaffolds, demonstrating that all 98.62% of mapped reads originate from the components assembled here rather than others unassembled.

**Table 1:** Species delineation for all components. FC2 and FC3 were delineated as *Clostridium straminisolvens* and *Brevibacillus borstelensis* respectively. Thus, these two genomes were used as references for the two components for reconstruction validation by TETRA, while the closest relatives were used as references for other components for reconstruction validation by TETRA

Sp.	# genomes^a^	Most closely related species	ANI (%)	AF (%)
FC1	7	*Clostridium clariflavum* 4-2a (GCA_000519985.1)	89.24	1.77
FC2	21	*Clostridium straminisolvens* JCM 21531 (GCA_000521465.1)	95.47	96.77
FC3	8	*Brevibacillus borstelensis* 3096-7 (GCA_000612185.1)	99.40	95.80
FC4	15	*Sedimentibacter* sp. B4 (GCA_000309315.1)	86.25	0.06
FC5	298	*Clostridium* sp. L74 (GCA_001276215.1)	91.88	74.93
FC6	0	NA	NA	NA
FC7	313	*Clostridium colicanis* DSM 13634 (GCA_001593985.1)	84.56	0.16
FC8	2	uncultured *Blautia* sp. (GCA_900066235.1)	71.04	0.16
FC9	4	*Atribacteria bacterium* SCGC AAA255-E04 (GCA_000398465.1)	0.00	0.00

^a ^genomes sieved by FRAGTE; *ANI *average nucleotide identity, *AF *Alignment fraction, *Sp. *species, *NA *not applicable

For the remaining reads, we performed the additional analyses as follows. First, our analysis by using the kmerfreq software [26] showed that approximately 97.11% of the unmapped reads were possibly sequencing errors, due to harboring 15-mers with ≤ 2 frequencies under such deep sequencing, leaving only 23,992 (~ 0.04% of total reads) unmapped reads with relatively high quality. Second, we assembled all unmapped reads to see whether there were other low-abundance organisms and found that only a total of 46,736 bp for 62 scaffolds (≥ 500 bp) was assembled, with a low N50 and maximum scaffold size of 731 bp and 2,015 bp respectively. It is worth pointing out that the complexity is greatly reduced from the total reads to the unmapped reads considering the complex nature of metagenomes with the coexistence of intragenomic or intergenomic repeats, which may generate some additional assemblies, some of which may be artificial due to excluding other reads. However, even under this simplified condition, the total size of assemblies is still greatly small, possibly indicating there are no low-abundance organisms in F1RT. Third, we mapped all the initially unmapped reads against the 62 scaffolds and found that 17,966 read pairs can be concordantly mapped back to their scaffolds. Further checking found that the 62 scaffolds used 8111 of the 23,992 high-quality (> 2 frequencies) reads as well as 9855 of the 806,187 low-quality (≤ 2 frequencies) reads, showing that the high-quality reads were used more significantly than the low-quality reads (*P* < 2.2e-16, the chi-squared test) for assembly, possibly indicating that the majority of low-quality initially unmapped reads are prone to sequencing errors and thereby cannot be successfully assembled even under this simplified condition. Fourth, although the 62 scaffolds may be with low credibility, we still performed alignment to explore their possible origins and found that 55 of them were successfully mapped to the F1RT metagenome reassembled here (Additional file 1), supporting that these scaffolds may originate from the components assembled here. For the remaining 7 scaffolds, we additionally BLASTed them against the nucleotide sequence database in the National Center for Biotechnology Information (NCBI) and found only 2 scaffolds were credibly (≥ 0.9 alignment fraction, AF) aligned, both of which are from the phylum Firmicutes. Further analysis showed that the mapping ratios of the initially unmapped reads against the references are only about 0.0272% and 0.0059% respectively (Additional file 2: Table S1), indicating that the 2 scaffolds are possibly not from the two references. For the remaining 5 unaligned scaffolds, they total only 3059 bp, possibly indicating there are not from the other low-abundance bacteria, as it is greatly impossible to assemble a such draft genome with a small size under so deep sequencing. Also, these 5 unaligned scaffolds may be greatly impossible from phages, as phages may have multiple copies in one cell and thereby should have high sequencing coverage. Therefore, all these results indicate that the possibility of containing other low-abundance organisms in F1RT is dramatically low, although we cannot completely exclude this possibility. However, all these results at least responsibly demonstrated that almost all F1RT MAGs were successfully assembled. As most of the binners use assemblies instead of reads for binning, directly using the entire F1RT metagenome reassembled here, without considering whether no low-abundance organisms exist, does not affect binning benchmarking below. Besides, all reads instead of just the mapped reads can be directly used to calculate sequencing coverage for all scaffolds *via* mapping them against the entire F1RT metagenome. However, we also deposited only all mapped reads at https://github.com/Yizhuangzhou/F1RT for users to accelerate mapping for convenience.

Using MetaGeneMark (version 3.38) [27], 31,775 genes (≥ 100 bp) had been totally predicted. All high-quality reads and original assemblies reused in this study were downloaded from the GigaScience database (http://gigadb.org/dataset/100049 ). For more details, please refer to our published study [21].

### Isolating, sequencing, and assembling 4 components

For the application of the binning assessment by reference to known genome assignments, almost all MAGs of F1RT should be constructed. So, we used serial dilution, plating, and repetitive subculturing to isolate its components and then determined colonies as axenic cultures by 16 S rRNA amplification and sequencing. Fortunately, we isolated 4 components in this study. Results from 16 S rRNA sequencing confirmed that these isolates are FC2-3, FC5 and FC7 respectively. Then, we performed genome sequencing at a sequencing depth of > 32 for them (Additional file 2: Table S2), ensuring *de novo* assembling them into draft genomes with high completeness ( > ~ 99.36%). All basic information about the assembled isolate genomes is tabulated in Additional file 2: Table S2.

### Reconstructing MAGs for all components

**Fig. 1:**
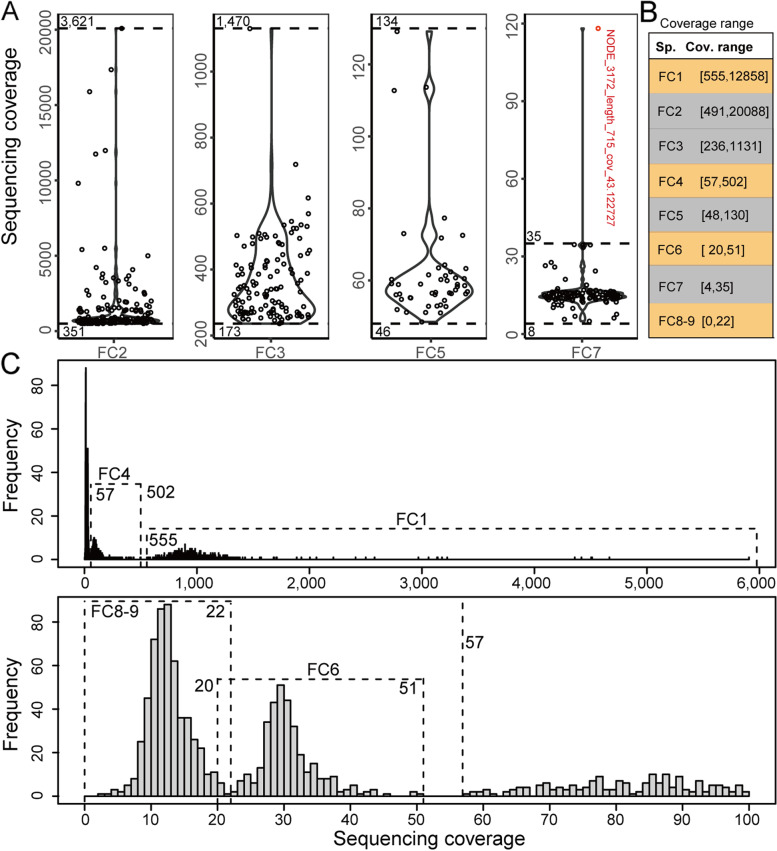
Sequencing coverage ranges determined for all F1RT components. **A **Distribution of scaffold-level sequencing coverage for each isolate. Red, scaffold with abnormally high sequencing coverage; scaffolds shown here are assembled by metaSPAdes. **B **Determined sequencing coverage ranges for all components. **C **Histogram showing the distribution of scaffold-level sequencing coverage for unaligned scaffolds assembled by SOAPdenovo. top, for all unaligned scaffolds; bottom, enlarged for unaligned scaffolds with average sequencing coverage < 100. Shown here is the average sequencing coverage across a whole scaffold

The general workflow is presented in Additional file 2: Fig. S1. It is noteworthy that all 4 isolates have > ~ 99.36% of assembly completeness (Additional file 2: Table S2), indicating that we could obtain their MAGs through individual genome alignment against the entire metagenome. By using the NUCmer tool (step 1, Additional file 2: Fig. S1) [28], we obtained alignment for each isolate (Additional files 3, 4, 5 and 6). It was observed that the vast majority of AFs are **≥** 90% (Additional file 2: Fig. S2), suggesting high credibility for their deduced relationships. Thus, scaffolds with AFs of **≥** 90% were preferentially untangled (step 2, Additional file 2: Fig. S1). All scaffolds were found to be individually mapped to one isolate genome and thus unambiguously deconvoluted, comprising the primary MAGs for the 4 isolates. Based on the primary MAGs, their sequencing coverage ranges at the whole-genome level were determined (Fig. 1 A and B). It is worth pointing out that one scaffold (indicated in red) harbors extremely high sequencing coverage (see Additional file 2: Fig. S10D below) possibly due to plasmid sequence (see Additional file 2: Table S5 below) and was thus excluded to determine the sequencing coverage upper bound for FC7.

The unaligned scaffolds are nearly pure for uncultured components for the following two reasons. One is > ~ 99.36% of assembly completeness for the 4 isolates (Additional file 2: Table S2). The other is that genome is more readily assembled for isolate than for metagenome, due to the complex nature of metagenome. We analyzed the sequencing coverage for all unaligned scaffolds reassembled by metaSPAdes and unfortunately found that there was no distinct separation (Additional file 2: Fig. S3). However, when using the unaligned scaffolds assembled by SOAPdenovo in the previous study [21], there are 4 distinct peaks (Fig. 1C), except for FC6 and FC8-9 (as indicated by two components below) with a minute fraction of overlappings (range of 20–22). It was shown that the majority of unaligned scaffolds uniquely assembled by metaSPAdes have < 20 or > 760 sequencing coverage (Additional file 7), and only need to extend the coverage range of FC1 to 12,858 without changing the coverage range for other components. By this means, 4 sequencing coverage ranges at the whole-genome level were determined (Fig. 1C), although sequencing coverage variations within FC1 and FC4 seem relatively large (~ 10×). As sequencing coverage follows a Poisson distribution [29], deep sequencing of 12.8 Gb reads for only 9 strains may result in larger intragenomic sequencing coverage variations for high-abundance FC1 and FC4 than for low-abundance FC5-9, demonstrated by the similar ~ 10× of intragenomic sequencing coverage variations for isolates FC2-3. In summary, the sequencing coverage ranges for all components were determined (Fig. 1B).

Unaligned scaffolds within only one sequencing coverage range were unambiguously untangled (step 3, Additional file 2: Fig. S1), comprising the primary MAGs for uncultured components. Once MAGs had been primed, models of all components could be trained. Based on the trained primary MAGs of FC6 and FC8-9, 2 scaffolds within the range of 20–22 were separated into FC6 by the Naïve Bayesian Classifier (4-nt motif) [30, 31], as tetranucleotide difference is generally larger between genomes than that within genomes [32].

Next, the scaffolds with < 90% AFs were deconvoluted (step 4, Additional file 2: Fig. S1). Scaffolds within only one sequencing coverage range were explicitly recovered into their MAGs, including 1 for FC4 and 3 for FC6, while the others were further untangled by using the naïve Bayesian classifier (4-nt motif) based on primary MAGs determined above [30]. A scaffold was flagged for a certain isolate if this isolate possessed the highest posterior probability, and meanwhile contained an alignment against this scaffold. Otherwise, scaffolds were determined to be from uncultivated species with a maximal posterior probability.

Our further SCG analysis showed that FC8-9 have two components, indicated by 54 SCGs with 2 copies and 2 SCGs with 3 copies (Additional file 8). FC8-9 have overlapping sequencing coverage and thus cannot be separated based on sequencing coverage. However, we, fortunately, found that the scaffolds harboring SCGs with > 1 copies are separated by using the posterior probability cutoff of -1350 determined by using the Naïve Bayesian Classifier with trained FC2 MAG (Additional file 2: Fig. S4). Similar patterns were found by using the trained FC3, FC5, or FC7 MAG (data not shown). Then, the primed SCG scaffolds were used as models to separate all scaffolds of FC8-9 (step 5, Additional file 2: Fig. S1).

Taken together, in this way (Additional file 2: Fig. S1), we reconstructed MAGs for almost all components. Statistics in terms of total numbers and total base pairs of untangled scaffolds in steps 1–4 are shown for both isolates (Additional file 2: Fig. S5) and uncultured components (Additional file 2: Fig. S6). Besides, it is worth stressing that two additional MAGs (hereafter termed FC8 and FC9) were strikingly discovered in this study, compared with the original study [21]. This finding explains the high redundancy of the original FC7 [21], as the original FC7 substantially contains three species (herein termed FC7, FC8, and FC9).

### Reconstruction validation based on SCGs

The vast majority of metagenomic scaffolds for 4 isolates were reliably untangled by comparison to isolate genomes with high credibility of **≥** 90% AFs (Additional file 2: Fig. S5), indeed requiring no additional validation. However, for comprehensive and solid validation on all uncultured MAGs even under the more complicated condition with isolates than without isolates, all MAGs were chosen to validate together.

We first validated reconstruction based on the 107 SCGs [33], which were previously applied to assess binning completeness and purity [34, 35]. A total of 907 SCGs were identified in the entire metagenome and then aggregated into scaffold level to identify 300 SCG-bearing scaffolds. By using these scaffolds, reconstruction was validated from the three following aspects independently.

**Fig. 2:**
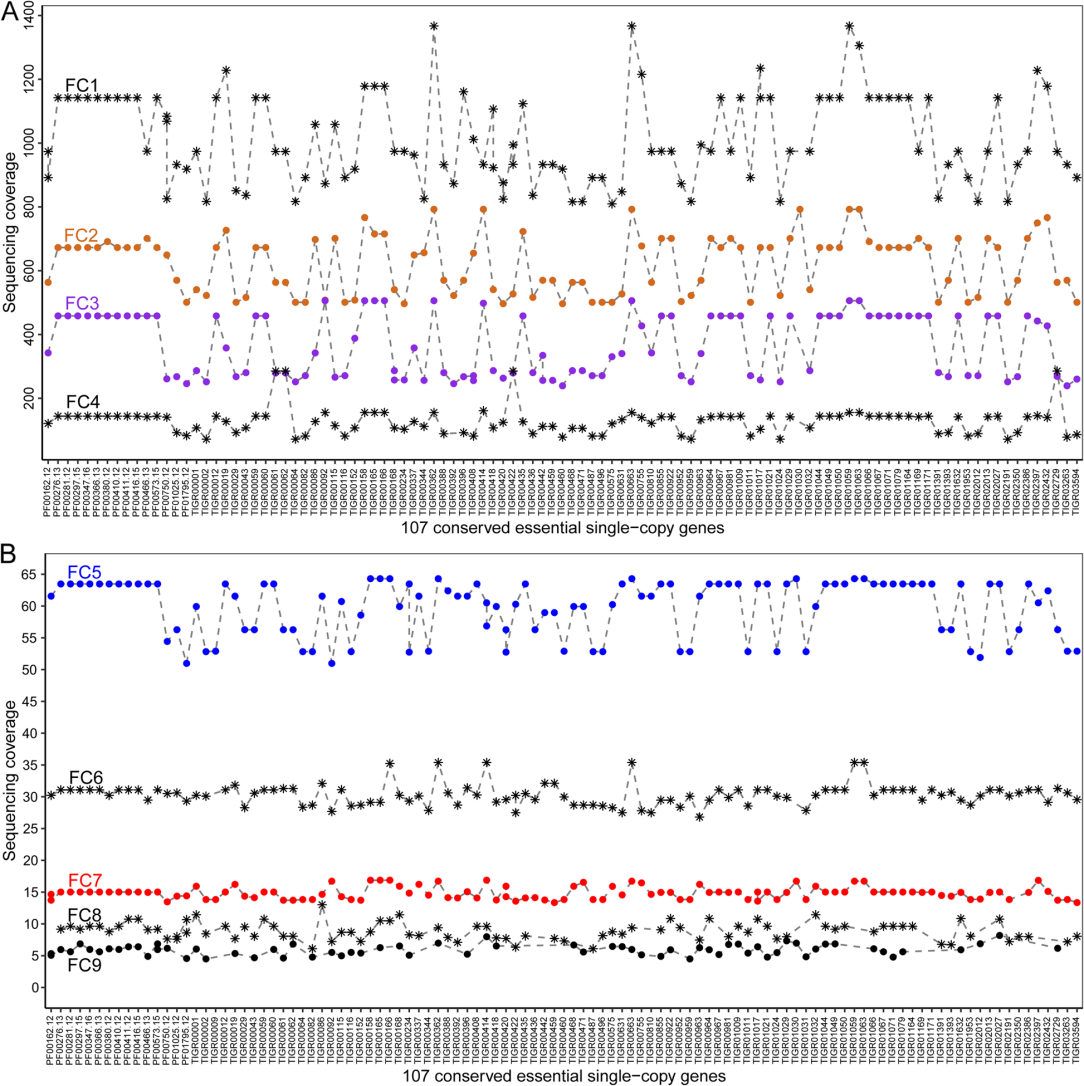
Reconstruction validation based on sequencing coverage of SCGs. **A **Sequencing coverage distribution for F1RT components FC1-4. **B **Sequencing coverage distribution for F1RT components FC5-9. Shown here is the average sequencing coverage across a whole scaffold

First, validation based on sequencing coverage showed that SCG-bearing scaffolds of the isolates separate all counterparts of uncultured organisms (Fig. 2), supporting that the above deduction of sequencing coverage ranges is reliable (Fig. 1C). Besides, the sequencing coverages for these scaffolds are gradually decreased from FC1 to FC9, indicating the high authenticity of our reconstruction.

**Fig. 3:**
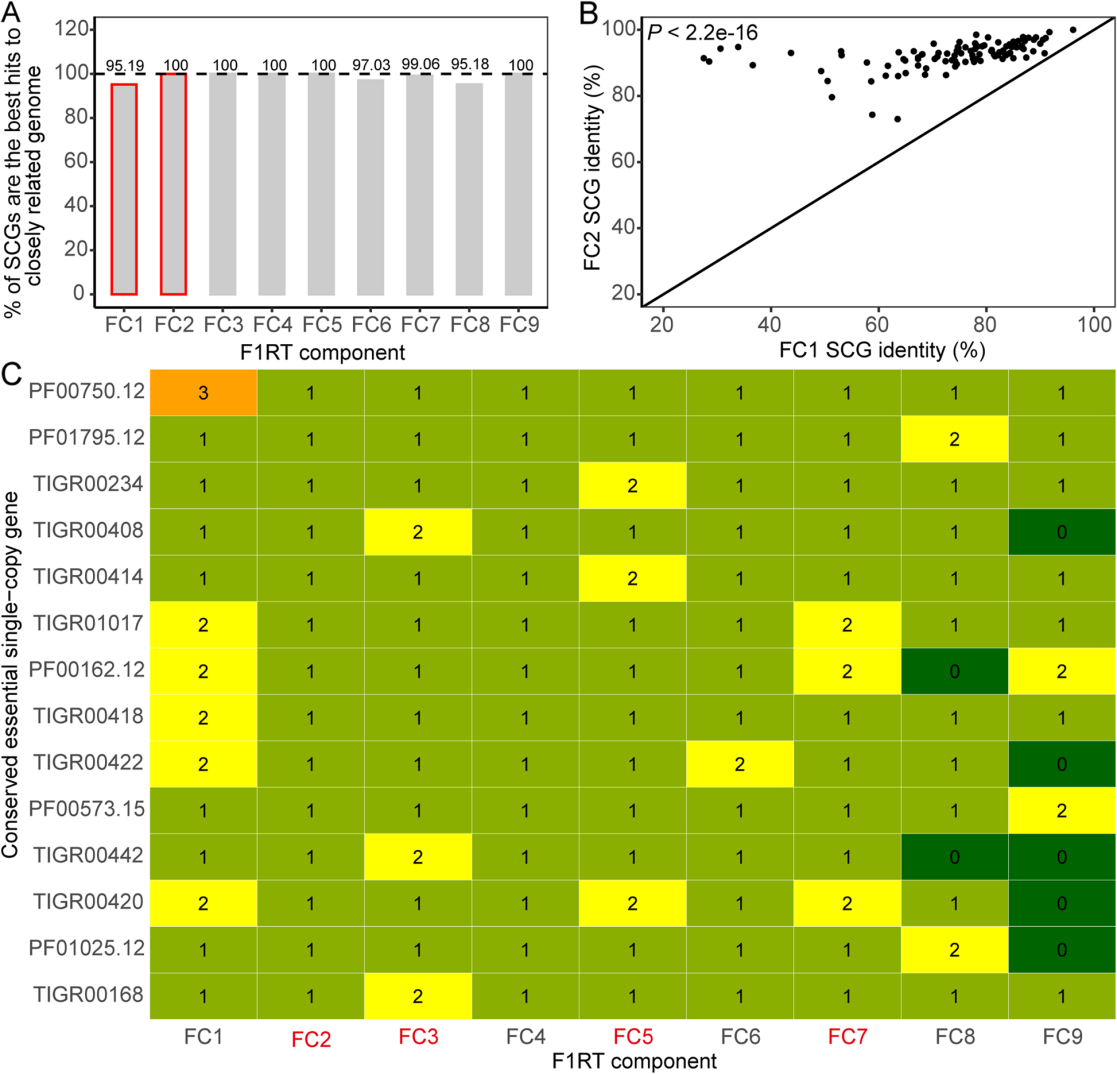
Reconstruction validation based on taxonomic annotation and single-copy feature of SCGs. **A **Percentage of SCGs which are best hits to close relatives. Red, the genome of *Clostridium* sp. Bc-iso-3 (GCA_001717745.1) used both for FC1 and FC2. **B **FC2 SCGs are more similar to counterparts of the close relative *Clostridium* sp. Bc-iso-3 than FC1 ones. The black line represents a straight-line y = x plot; *P*-value, paired Wilcoxon signed rank test. For reference genomes, see Additional file 2: Table S3. **C **Validation based on the single-copy feature. The number in the cell is the amount of a certain SCG and is used as a basis for color intensity. Red, for isolate

Second, taxonomic analysis was used based on the logic that SCGs originating from the same genome should be taxonomically concordant [36]. A total of 21,124 selected references (for details, see Methods) were used to search for their closest relatives. The reference with the highest SCG-based average amino acid identity (AAI) (termed SCG AAI) was determined as the closest relative (Additional file 2: Table S3). Then, we performed alignment of SCGs between each MAG and all closest relatives and found that for almost all components except for FC1, the SCG AAI distance between the closest relative and the reference with the second highest SCG AAI is very large (> 10.98) (Additional file 2: Fig. S7), while the SCG AAI distance for FC1 is only 1.12. It was reported that FC1 is a strain possibly from *Clostridium* sp. FG4 (16 S rRNA identity 99.3%) or *Clostridium thermosuccinogenes* (16 S rRNA identity 99.1%) [21]. However, genomes for these two strains are unavailable yet. Therefore, both closest relatives for FC1 (*Clostridium josui* JCM 17,888) and FC2 (*Clostridium* sp. Bc-iso-3) (Additional file 2: Table S3) were jointly employed to count the number of best hits for FC1. We found that almost all (> 95.18%) SCGs are taxonomically concordant (Fig. 3A), manifested by the fact that almost all best hits are concurrently from one close relative for FC2-9 or the combination of *C. josui* JCM 17,888 and *C.* sp. Bc-iso-3 for FC1 (Additional file 2: Fig. S8). Further analysis showed that 100% of FC2 SCGs are concurrently and significantly more similar to SCGs of *C.* sp. Bc-iso-3 than those for FC1 SCGs (Fig. 3B), implying low or even no contamination for FC1 and FC2.

Third, validation based on the single-copy characteristic showed that MAGs were reconstructed to the theoretical limit of contamination, as almost all SCGs with > 1 copy except for PF00162.12 and PF01025.12 for FC8-9 are true duplication rather than contamination (Fig. 3C and Additional file 9). As all components are from the phylum Firmicutes (Additional file 2: Table S3), we further analyzed all 27,565 Firmicutes genomes and showed that almost all SCGs with > 1 copies except for TIGR00442 are veritably frequent with > 1 copies (median 0.52 for the percentage of genomes with > 1 copies) (Additional file 2: Fig. S9A). However, it is worth pointing out that only 4.73% of analyzed Firmicutes genomes are complete (Additional file 2: Fig. S9B) and about half of them harbor < 107 SCGs (Additional file 2: Fig. S9C), meaning that the possibility to be multiple may be underestimated here. Taken together, an assessment based on SCGs strongly indicated that our reconstruction is very reliable.

### Reconstruction validation based on the whole-genome sequencing coverage

As conserved essential SCGs are located in a restricted part of a genome, validation based on SCG scaffolds estimates the reconstruction roughly. For elaborate validation, we extended SCG-bearing scaffolds to the whole MAGs. Next, we verified the MAG reconstruction based on sequencing coverage at the whole genome level. To alleviate scaffold size-induced unevenness, the coverage used here was calculated by using a fixed window of 500 bp with 250 bp overlap, while the above analyses (Figs. 1 and 2) were based on the average coverage across a whole scaffold. Our logic was that an idealized MAG should have only one main peak for sequencing coverage and MAG failing this condition probably indicates somehow contamination. Our results showed that both isolates (Additional file 2: Fig. S10) and uncultivated components (Additional file 2: Fig. S11) individually have only one main peak, providing evidence that our reconstruction is very confident.

### Reconstruction validation based on the whole-genome nucleotide composition

**Fig. 4:**
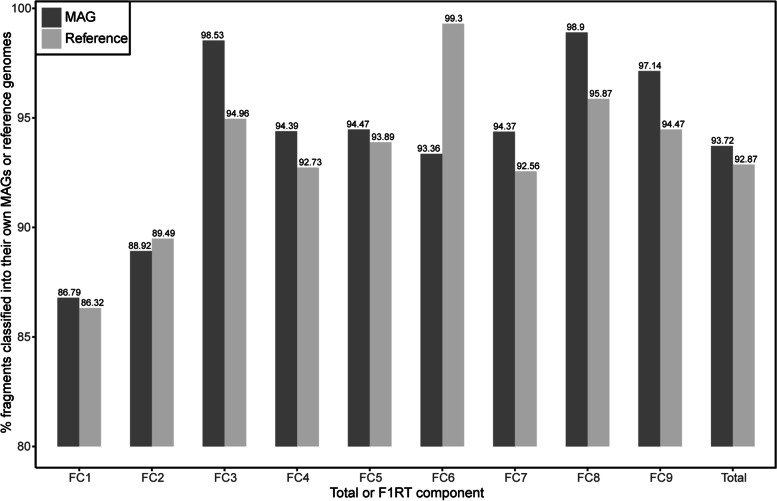
Reconstruction validation based on the whole MAG composition by TETRA. For references, please refer to Additional file 2: Table S4

It has been extensively reported that intragenomic oligonucleotide composition is generally more homogenous than intergenomic one [32, 37, 38]. Consequently, oligonucleotide composition was applied for reconstruction validation. Here, tetranucleotide frequencies were used as oligonucleotide composition considering its great balance between distinguishing ability and computing cost [39]. Besides, we previously found that the TETRA method could represent other statistic methods for tetranucleotide frequency [39] and thus TETRA was employed in this study. Reconstruction reliability is assessed by classification accuracy by TETRA. We found that ~ 86.79% for FC1 to ~ 98.90% for FC8 were correctly classified back into their MAGs with a total high 93.72% of classification accuracy (Fig. 4), proving that a vast fraction of scaffolds was correctly reconstructed. For comparison, we further performed an identical analysis for their references (Additional file 2: Table S4), including two species-level references for FC2 and FC3 (Table 1, see below) and six close relatives based on SCG AAI for FC1 and FC4-9 without species-level references (Additional file 2: Table S3). We found that classification accuracies for MAGs are even larger than those for references for all except for two isolates FC2 and FC6 (Fig. 4), yielding a larger total classification accuracy for MAGs than that for references. These results demonstrated that our reconstruction is pretty authentic. Besides, it has been reported that the oligonucleotide composition of closely related organisms is more similar than that of distantly related organisms [40], namely that a larger portion of fragments may be inherently misclassified between closely related organisms than between distantly related species. Expectedly, detailed tracking showed that a large fraction of fragments is substantially misclassified into closely related organisms. For example, ~ 9.79% of FC1 fragments were incorrectly classified into FC2 (Additional file 2: Fig. S12A), which is most closely related to FC1 (Additional file 2: Fig. S13A). Similar results were obtained for corresponding references (Additional file 2: Fig. S12B and Fig. S13B). These results imply that reconstruction reliability may be underestimated here. In addition, it is worth pointing out that the classification accuracy for FC2 MAG is slightly lower than that for its reference, while although the classification accuracy for FC6 MAG is relatively lower than that for its reference, its accuracy is still high (~ 93.36%) (Fig. 4).

### Properties suitable for benchmarking binning

**Fig. 5:**
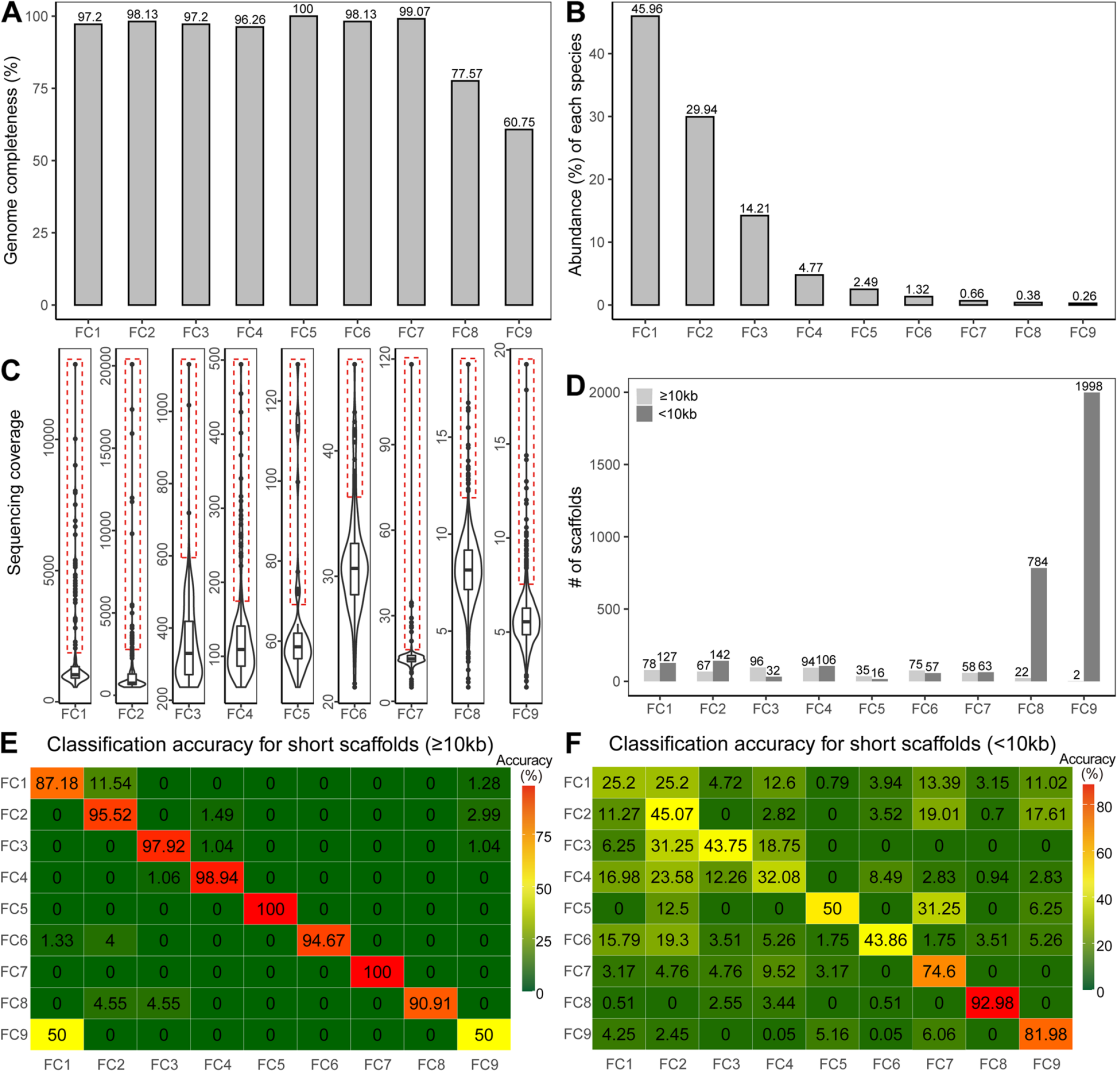
Properties with suitability as a benchmark. **A **Genome completeness. Genome completeness is the number of kinds of SCGs divided by 107. **B **Abundance for each component. **C **Sequencing coverage distribution for each component. boxed, abnormally high sequencing coverage. **D **Length distribution for scaffolds. **E **Classification accuracy for long scaffolds. The classification was run by comparing the TETRA values based on the whole reconstructed MAGs. **F **Classification accuracy for short scaffolds. The classification was run by using the naïve Bayesian classifier with training the whole reconstructed MAGs

Genome completeness, which was roughly assessed as the number of SCGs divided by 107 owing to about half of Firmicutes having 107 SCGs (Additional file 2: Fig. S9C), ranges from 60.75 to 100% (Fig. 5A), supplying an opportunity to assess binning performance for samples with a broad range of assembly completeness. It is worth stressing that a binned genome with low completeness may result from relatively low abundance rather than bad binning, such as FC8-9 here. In this context, for binning evaluation, the strategy by comparison to known genomes outperforms the strategy with reliance on SCGs. Besides, the relative abundances vary substantially, including ~ 45.96% for dominant taxa FC1 and even < 1% for rare components FC7-9 (Fig. 5B), providing a possibility to benchmark binners for both high- and low-abundance organisms.

Collectively, three main discriminative characteristics underlie the binning tools, including reference (known) genomes, sequencing coverage (abundance), and sequence composition. The relatively outdated binners leverage one of them alone [32, 41–44], while the state-of-the-art binners at present leverage two or more characteristics together [12, 20, 45–52]. Here, we independently investigated them to see whether this dataset can be well binned by using one of them alone.

For known genomes, our results showed that only FC2 and FC3 possess species-level references (Table 1), according to the ~ 95% average nucleotide identity (ANI) threshold for species delineation [53]. As a result, it is impossible to deconvolute the whole metagenome by merely using reference genomes, in line with the condition for most metagenomic studies with a paucity of reference genomes.

For sequencing coverage, we found that it was impossible to separate any MAGs (Fig. 5C), if not using any other information such as isolates in this study. In addition, we observed that there is a small portion of scaffolds with abnormally high sequencing coverage (Fig. 5C), greatly challenging for binners (For reasons, see Discussion below). In contrast, datasets generated through in *silico* simulating sequencing reads possibly have no such characteristics.

For sequence composition, binning accuracy is low for short scaffolds (< 10 kb) [32, 54]. It is worth pointing out that short scaffolds are differently defined from other studies. For example, CONCOCT and MetaBAT2 defined < 1-2.5 kb sequences as short scaffolds [20, 47], as < 1 kb sequences are unable to accurately capture species-specific composition and coverage patterns. This difference is possibly because these binners use sequencing coverage together with composition to improve binning. We discovered that the vast majority of scaffolds are < 10 kb for FC8-9 (Fig. 5D), posing a substantial challenge to binners solely based on composition. Furthermore, we found that still a fraction of long scaffolds (≥ 10 kb) are misclassified (Fig. 5E), even under the condition using the whole above-reconstructed MAGs (for details, see Methods), which yield higher classification accuracy than under the condition by using individual scaffolds during the binning period when their whole MAGs are indeed unknown [54]. Besides, it was reported that the naïve Bayesian classifier is suitable to classify short scaffolds with even < 1000 bp [30], although it has not been successfully integrated into binning methods yet. Here, we explored whether short scaffolds can be correctly classified with the naïve Bayesian classifier based on the trained whole MAGs (for details, see Methods) and found that the binning accuracies for short scaffolds are only ~ 25.20-92.98% (Fig. 5F), also showing challenging for binning.

Together, these findings indicated that F1RT albeit simple is amazingly challenging for binners based on one characteristic alone, requiring state-of-the-art binners which leverage several characteristics [12, 20, 45–52]. Thus, the dataset constructed here is a pretty good benchmark to compare all binners including mainstream binners available now or assess new binners leveraging several characteristics.

### Evaluating metagenomic binners with the constructed dataset

**Fig. 6:**
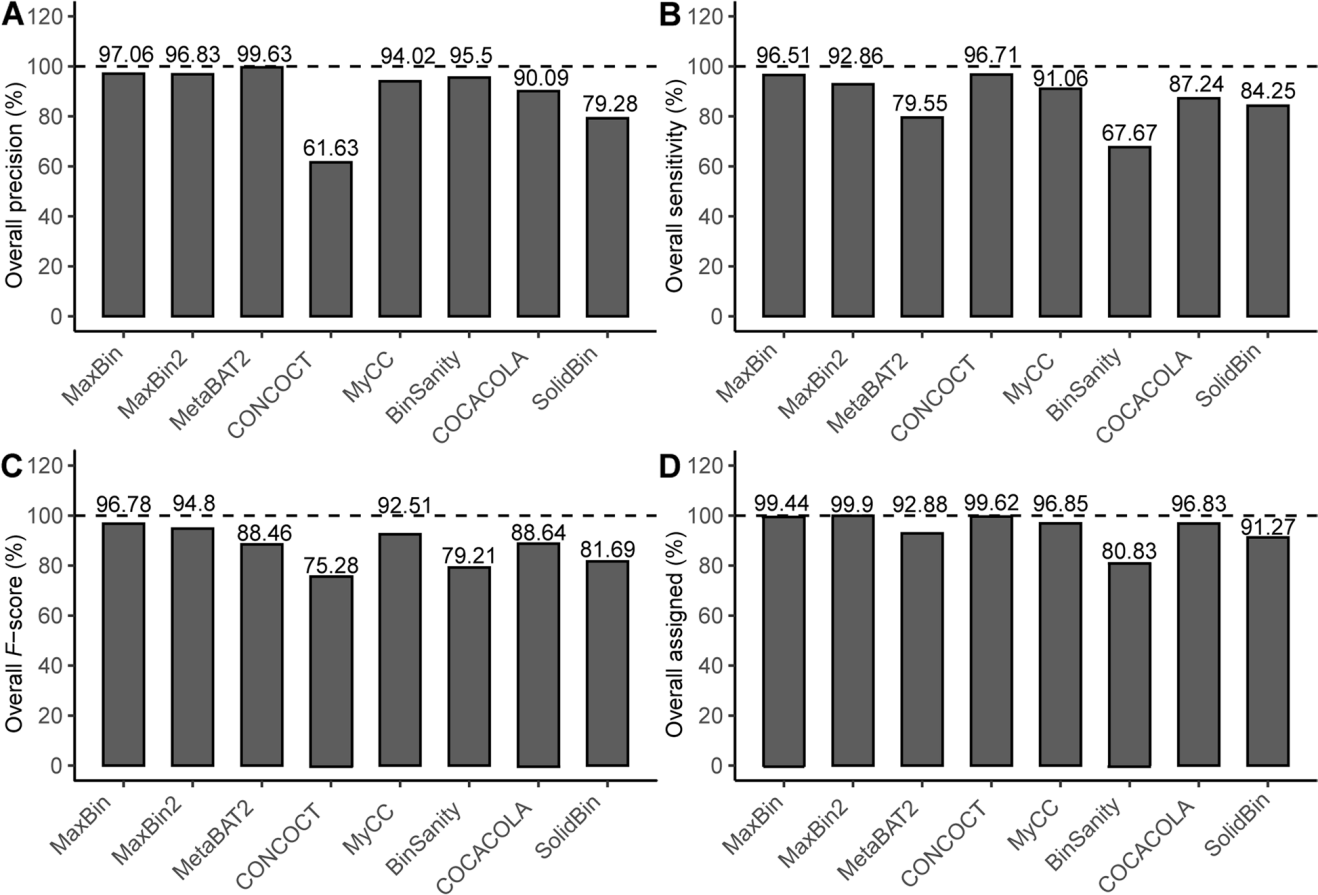
Binning performance of 8 binners on F1RT dataset. **A **Overall precision; **B**, overall sensitivity; **C **overall *F*-score; **D **overall percentage of recovered base pairs. Dashed line, gold standard

**Fig. 7:**
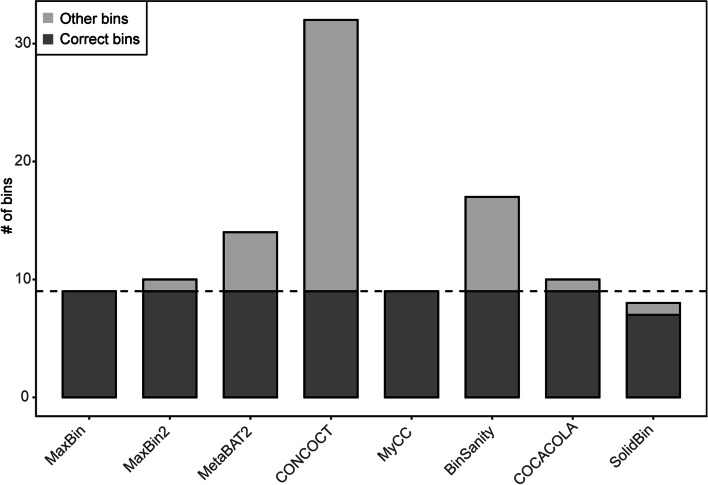
The number of bins for 8 binners on the F1RT dataset. Correct bins, clusters satisfying $\mathop {{\text{max}}}\limits_{i} (\mathop {\hbox{max} }\limits_{j} {R_{ij}})$ (see Methods for details); other bins, clusters not satisfying $\mathop {{\text{max}}}\limits_{i} (\mathop {\hbox{max} }\limits_{j} {R_{ij}})$; dashed line, gold standard of 9 components

Ensemble binning tools can be separated into two classes: (1) the stand-alone binners such as MaxBin [12] and CONCOCT [20]; (2) the binners refining results of other binners, such as GraphBin [55], GraphBin2 [56], METAMVGL [57] and MetaWRAP [58]. Here, we used this constructed dataset to assess the 8 aforementioned first-class stand-alone binners (for details, see Methods). MetaBAT2 achieved the highest overall precision (99.63%), followed by MaxBin and MaxBin2 with comparably good precision (Fig. 6A). Nonetheless, MetaBAT2 showed relatively low sensitivity of 79.55% (Fig. 6B), while MaxBin and MaxBin2 produced a higher sensitivity of 96.51% and 92.86% respectively. Accordingly, MaxBin, followed by MaxBin2 and MyCC, generated the best aggregate binning performance, as indicated by *F*-scores (Fig. 6C). In addition, MaxBin, MaxBin2 and CONCOCT recovered almost all (> 99%) scaffolds (≥ 500 bp) (Fig. 6D), while MetaBAT2 recovered a relatively lower fraction (92.88%), implying that MetaBAT2 may only bin scaffolds with high accuracies to generate the highest precision (Fig. 6A), as MetaBAT2 does not bin < 2.5 kb scaffolds [47, 59]. It is worth pointing out that CONCOCT yielded the lowest precision to achieve the minimal *F*-score. However, it may be a fair comparison for CONCOCT if multiple (usually > 50) samples are used [20]. It is also worth noting that the performance of MetaBAT2 was severely impaired without multiple samples [47]. Also, BinSanity, SolidBin, and COCACOLA met the obstacle to yield an *F*-score of > 90%, although they were developed later than MaxBin. Besides, we found that only MaxBin and MyCC recovered the correct number of 9 bins (Fig. 7), while SolidBin only correctly yielded 7 bins with one additional incorrect bin. Other methods split MAGs into multiple bins to yield large bin numbers. Together, MaxBin performed best on the F1RT dataset constructed here.

## Discussion

### Possible reasons for abnormally high sequencing coverage

Our results showed that some scaffolds harbor abnormally higher sequencing coverage than other scaffolds in their MAGs (Figs. 1 and 5C, and Additional file 2: Fig. S10 and S11). For explanation, we performed the following additional analysis and obtained some clues. First, we found no significant difference in mapping qualities for scaffolds with abnormally high sequencing coverage compared to scaffolds with normal sequencing coverage (Additional file 2: Fig. S14), indicating that mapping quality is not the main reason for F1RT. The decreased mapping quality may result from randomly low-quality sequencing, which may be nonselective for any scaffolds in the same MAG, explaining the above observation. As duplicated reads generated by PCR amplification before sequencing or during the period of next-generation sequencing like Illumina sequencing used here may generate high sequencing coverage, we next explored this possibility and expectedly found that some scaffolds with high sequencing coverage indeed have relatively higher duplication than normal ones (Additional file 2: Fig. S15), possibly accounting for their higher sequencing coverage. As plasmids may have multiple copies in cells to generate higher sequencing coverage than chromosome sequences, we then performed alignments between plasmid sequences and scaffolds with high sequencing coverage and found that some are possibly from plasmids (Additional file 2: Table S5). Finally, our results showed that some scaffolds with high sequencing coverage are shorter than normal ones (Additional file 2: Fig. S16), indicating that they may localize at intragenomic or intergenomic regions, as repeats are difficult to be assembled. However, the exact reasons underlying abnormally high sequencing coverage for some scaffolds remain unknown yet, and whether existing other reasons awaits further research.

### Implication for future investigation on microflora

Despite great progress in culture technologies [60], uncultivability for the overwhelming majority of bacteria remains a major challenge yet. However, some component species could be isolated elaborately in pure culture. Sequencing the obtained monospecific colony will provide a fraction of isolate genomes, which in return efficiently reduces the complexity of the original target metagenome. Here, we demonstrate an effective strategy, namely that subtracting isolate genomes from the entire genome could untangle genomes for uncultured organisms. However, although pure cultures maybe not be easily obtained in some conditions, simplified mixed cultures may be roughly obtained. Miniature microflora will provide mini-metagenome, which in return similarly reduces the complexity of the original target metagenome to facilitate binning [61]. Therefore, it is evident that a combination of culturing regardless of pure culture or simplified mixed culture and classical metagenomics will increase the quality and quantity of genomes recovered.

### Using F1RT as a benchmark of single metagenome

Combining multiple samples for binning increases the dimensionality of sequencing coverage to improve binning [35, 62]. However, it also possesses several limitations. First, components, which are unique to one sample [63], will not necessitate multiple-sample sequencing. Second, it incurs high sequencing costs. Third, it requires elevated computational resources to co-assemble reads from multiple samples. Forth, the co-assembly required for multi-sample algorithms diminishes the strain-level microdiversity [35], which may be indispensable for functional investigation [64]. Therefore, in these circumstances, single metagenomes necessitate maximizing usable information. As F1RT is a single metagenome, it can serve as a particularly helpful toolkit for the realistic assessment of performance on single-sample metagenomes for binning methods and exploring whether available methods make full use of leveraging information without requiring additional samples. Besides, we should point out that if a tool works well on F1RT, it could potentially work well on complex metagenomes (because this cannot be guaranteed every time, especially if the metagenome has closely related strains with similar abundance). However, a tool, which yields low performance on F1RT, is surely not good enough and requires further improvement.

### Insights from the benchmarking of 8 binning tools

Almost all mainstream binners available now use an ensemble of sequence composition and sequencing coverage (including both single and multiple samples) for binning. MaxBin2, albeit using the multiple-sample abundances, performed slightly less accuracy than MaxBin (Figs. 6C and 7), implying that MaxBin2 makes poor use of single sequencing coverage. Therefore, we reason those tools using multiple-sample abundances should primarily make full use of single-sample abundance. Besides, some tools developed recently apply some additional information. For example, COCACOLA uses pair-end read linkage [50]; SolidBin applies co-alignment information including must-link and cannot-link constraints [51]. However, our findings showed that SolidBin and COCACOLA obtained lower *F*-scores than MaxBin (Fig. 6C) and yielded incorrect bin numbers (Fig. 7), indicating that they make poor use of sequence composition and sequencing coverage or poorly combine additional information with the basis of sequence composition and sequencing coverage. Therefore, novel tools should first make full use of sequence composition and sequencing coverage as a base and then integrate other information such as co-alignment information, pair-end read linkage, SCGs, assembly graphs [65], and DNA methylation [66] to further improve binning, or assembly graphs [55, 56] and a combination of assembly and paired-end graphs [57] to further refine binning of initial binners such as MaxBin2 [56, 57].

### The significance of the application of F1RT MAGs

When developing a novel algorithm, reiterative assessment regarding the strengths and limitations and accordingly improvement/optimization is required. Hence, a simple dataset with < 10 species is greatly beneficial and convenient, as it can reduce the runtime for each test. Thus, F1RT is an ideal investigative model, due to its embracing only 9 components. In addition, the dataset constructed here is the first dataset with all known genomes for a real consortium. Compared with simulated datasets, this dataset provides some distinct characteristics challenging for binning. For example, a minute fraction of scaffolds with abnormally high sequencing coverage and low completeness for some components like FC8-9 here were found in F1RT. Therefore, a dataset like F1RT is indispensable.

Lots of advanced algorithms incorporate SCG information to improve binning, albeit with a slight usage difference. For example, COCACOLA [50], MaxBin [12], and SolidBin [51] use SCGs to estimate the initialized bin number; MyCC [48] and MetaWatt [41] use SCGs to determine which clusters should be merged or split after a round of clustering; Autometa uses SCGs to guide clustering [67]; MetaBinner uses SCGs for k-means initialization [68]. In this scenario, using SCGs for binning assessment may be unreasonable and unfair. As an alternative, F1RT could be used to evaluate these methods owing to known genomes for all components. Accordingly, F1RT can complement simulated datasets like CAMI datasets [13, 14] to complete a full assessment for all binning tools.

According to the above discussion, F1RT can be used as a benchmark for binning tools like MaxBin based on a single metagenome. As tools based on multiple metagenomes have evolved from the tools based on single metagenome [35] and single sequencing coverage is the base for developing multiple-metagenome-based tools, F1RT can be used as a benchmark to assess whether they make full use of single metagenome. In all, as a thoroughly independent and in-depth benchmark, we envisage that this dataset will become a standard dataset for comparison and improvement of binning methods in the future.

## Conclusion

Here, through isolating 4 components and then using the in-house developed pipeline to construct almost all genomes with 4 isolates and the whole metagenome, we present the first dataset with known genomes for almost all components in a real bacterial consortium F1RT. Besides, compared with the original study, two novel components termed FC8 and FC9 in this study are discovered and 7 reliable genomes for species without available genomes are obtained. This dataset has several features suitable for binning benchmarking and has wide applications including (*i*) benchmarking each round of improvement when developing novel tools owing to its low complexity; (*ii*) comparing state-of-the-art tools available as an independent benchmark; (*iii*) as a base for exploring whether tools make full use of single-sample sequencing coverage to develop robust tools and (*iv*) assessing tools with the integration of SCGs for binning. Besides, this study demonstrates an effective strategy *via* combining culturing and classical metagenomics for uncovering uncultured genomes. Finally, this study provides useful insights for developing more promising tools. In all, this dataset will become a standard dataset for binning assessment on a real consortium and facilitate metagenomics in the future.

## Methods

### Isolation, growth conditions of F1RT isolates

Pure cultures were obtained from single colonies by iterative subcultivation on the plates and identified by analyzing 16 S rRNA gene sequences. FC2 was putatively determined to be *Clostridium straminisolvens* (16 S rRNA identity 99.6%) according to the 16 S rRNA identity threshold of 98.65% [69]. Thus, FC2 was isolated and cultured at 50 ℃ in DMSZ medium 122 in anaerobic conditions according to the previous study [70]. Besides, FC3 was isolated and cultivated in DMSZ medium 1 for DSM 6347 at 37 ℃ in aerobic conditions; FC5 and FC7 were isolated and cultivated in DMSZ medium 122 and DMSZ medium 1 for DSM 6347 respectively at 37 ℃ in anaerobic conditions.

### Genome sequencing and assembly of F1RT isolates

DNA was extracted based on the CTAB method. Illumina DNA PCR-free libraries with an insert size of ~ 500 bp were constructed for all 4 isolates according to the manufacturer’s instructions. Besides, a library with an insert size of ~ 2000 bp was additionally prepared for FC2. DNA manipulations, including the preparation of single-molecule arrays, cluster growth, and paired-end sequencing, were performed using an Illumina HiSeq 2000 sequencer according to standard protocols. The Illumina base-calling pipeline (version HCS1.4/RTA1.12) was used to process the raw fluorescent images and call sequences. Raw reads of low quality (those with three consecutive bases with quality ≤ Q20) were discarded before assembly. High-quality reads were assembled using the genome assembler SOAPdenovo2 with default parameters [71].

### Comparison of isolate genome and metagenome

The NUCmer tool (version 3.23) [72] was utilized to perform genomic alignment against the whole metagenome for each isolate genome (-maxmatch). Then, the utility delta-filter was employed to filter NUCmer output (-q –r –l 200), and the show-coords utility was used to generate the aligned coordinates (-c –l –r –T). Finally, a custom Perl script in-house was used to calculate AFs.

### Sequencing coverage determination

High-quality reads retrieved from the original report (http://gigadb.org/dataset/100049 ) were mapped back onto the F1RT metagenomic scaffolds to determine coverage with SOAPaligner (v. 2.21) [73] by allowing at most two mismatches in the first 35-bp seed region and 90% of identity over the whole read. Subsequently, base coverage was calculated by SOAPcoverage (v. 2.7.7). It was shown that the sequencing coverage at both ends of each scaffold is slightly lower than in other regions. To obviate the effect of scaffold ends, both ends of 100 bp were discarded. Then the average coverage across the whole scaffold was computed to represent the sequencing coverage of this scaffold. For drawing Fig. S10 and Fig. 11 in Additional file 2, the sequencing coverage was calculated from 5’ to 3’ end with a window of 500 bp and sliding at 250 bp.

### Determination of scaffolds by the naïve bayesian classifier

For scaffolds with multiple possible origins during the MAG reconstruction period (Additional file 2: Fig. S1), the naïve Bayesian classifier (4-nt motif) [30] was applied. Briefly, the primed MAGs were trained. Then, each scaffold was classified into MAG with the highest posterior probability.

### Identification of essential SCGs

A set of 107 Hidden Markov Models for SCGs [33] deposited in either TIGRFAMs [74] or Pfam libraries [75] were searched against the protein sequences of F1RT using HMMER3 with the default settings, except the trusted cutoff was used (-cut_tc). When one gene had multiple SCG annotations, only the one with a minimum e-value was assigned.

### Collection of reference genomes for SCG taxonomic analysis

All 83,075 prokaryotic genomes were downloaded from the NCBI database. To filter out low-coverage genomes, 37 draft genomes with a summed length < 0.5 megabase pairs were discarded. Then based on the List of Prokaryotic names with Standing in Nomenclature database, 68,261 genomes were determined for 5680 named species. The remaining 15,444 genomes were directly as reference genomes. For each named species, one type strain (if available) or the largest genome was selected as the reference. In total, 21,124 genomes were used as references for SCG taxonomic analysis.

### Reconstruction validation by taxonomic analysis of SCGs

SCGs were identified for the resulting 21,214 references. SCGs in MAGs were then BLASTPed against the SCG database with a maximum e-value cutoff of 1e-5 and the resulting identities were transferred into protein-length-weighted sequence identities. Then, SCG AAI was calculated for each MAG. To ensure high confidence, > 95% SCGs should be mapped between the compared genomes. The close relatives with the highest SCG AAIs were thus found (Additional file 2: Table S3). Best hits from close relatives were counted to validate reconstruction.

### Reconstruction validation by the single-copy characteristic of SCGs

Our results showed that all components are from the phylum Firmicutes (Additional file 2: Table S3). Thus, all 27,565 Firmicutes genomes downloaded from NCBI were subject to SCG identification. Then, the number of genomes with > 1 copy of each SCG was independently counted. Also, the assembly statuses and the number of SCGs across a genome were counted. All were tabulated in Additional file 2: Fig. S9.

### Reconstruction validation by TETRA

For each MAG, all its scaffolds were sorted decreasingly according to their sequencing coverage and then concatenated directly. Subsequently, all MAGs were shredded into consecutive 10-kb fragments with 5-kb overlap. For each fragment, 9 TETRA values against all MAGs were calculated according to the previous studies [32, 39, 54] and the MAG with the highest TETRA value was assigned for this fragment. The percentage of fragments correctly classified back into their own MAGs was counted to indicate the reconstruction performance. Also, an identical analysis was performed for their references listed in Additional file 2: Table S4.

### Species delineation for F1RT MAGs

The FRAGTE method [54] was used to sieve closely related genome pairs from 9 × 21,124 pairs for species delineation. Then we calculated ANIs for all sieved pairs by using the NUCmer tool (version 3.23) according to the previous study [53]. MAG with an ANI of > ~ 95% was delineated at the species level, according to the ANI cutoff determined previously [53].

### Classification of short scaffolds by the naïve bayesian classifier

Short (< 10 kb) scaffolds were classified by the naïve Bayesian classifier (4-nt motif) [30]. Briefly, the whole MAGs were trained. Then, each short scaffold was classified into MAG with the highest posterior probability. The percentage of scaffolds classified back into their own MAGs was counted to indicate the classification accuracy.

### Binning benchmarking on the herein constructed dataset

Hybrid methods, which at least jointly leverage both sequence composition and sequencing coverage, are the most advanced. F1RT has only 3,852 scaffolds, while Vamb requires > 50,000 sequences for binning [76]. Therefore, Vamb was not benchmarked in this study. Also considering that F1RT is a single metagenome, algorithms such as GroopM [49] and MetaBMF [77] requiring multiple samples were excluded. This rationale led us to focus on the 8 first-class stand-alone binning tools without human-augmented refining, including MaxBin (v.1.4.5) [12], MaxBin2 (v. 2.2.7) [46], MetaBAT2 (v. 2.12.1) [47], CONCOCT (v.0.4.0) [20], MyCC (v.1.0) [48], Binsanity (v.0.5.4) [52], COCACOLA (v. 1.0) [50] and SolidBin (v.1.2) [51]. Here, Binsanity used the mode of Binsanity-lc comprising of binsanity and binsanity-refine, and SolidBin used the mode of SolidBin-naive. All used the default parameters except for the minimal length of 500 if allowed.

### Evaluation metrics

Assume there are *N* genomes in the dataset, which were assigned into *M* bins, and *S*_*ij*_ indicates the total sequences (in terms of base pairs) belonging to genome *j* appear in cluster *i*. If one bin has ≥ 2 assigned species, only the species with the largest number of sequences are kept for this bin and denoted as $\mathop {\hbox{max} Sij}\limits_{j}$; if one genome is assigned into ≥ 2 bins, only the bin with the largest number of sequences is kept for this genome and denoted as $\mathop {\hbox{max} Sij}\limits_{i}$. Furthermore, if ≥ 2 bins are assigned to one common species, the bin with the largest number of sequences for this species is denoted as $\mathop {{\text{max}}}\limits_{i} (\mathop {\hbox{max} }\limits_{j} {R_{ij}})$. Then only bins satisfying $\mathop {{\text{max}}}\limits_{i} (\mathop {\hbox{max} }\limits_{j} {R_{ij}})$ are considered correct bins, while others are considered incorrect bins. The overall precision and sensitivity, as in the previous studies [12, 48], were calculated as follows:

$$Precision=\frac{{\sum\limits_{i-1}^M}{\underset{j}{\max}}\; S_{ij}}{{\sum\limits_{i=1}^M}{\sum\limits_{j=1}^N}S_{ij}}$$

$$Sensitivity=\frac{\sum\limits_{{j=1}}^{N} {\mathop {\hbox{max} Sij}\limits_{i} } }{\sum\limits_{{i=1}}^{M} {\sum\limits_{{j=1}}^{N} {Sij+\sum {{\text{unbinned sequences}}} } } }$$

*F*-score, which indicates the overall binning performance *via* weighting both overall precision and sensitivity, was calculated as follows:

$$F-score=\frac{2\times\left(Precision\times Sensitivity\right)}{Precision+Sensitivity}$$

## Supplementary Information

Additional file 1.  Coordinates for nucleotide-based alignments between scaffolds assembled by unmapped reads and F1RT metagenome scaffolds (F1RT) reassembled by metaSPAdes.

Additional file 2. **Figure S1.** A schematic flow chart for untangling MAGs for almost all F1RT components. Steps including genome-wide alignment by NUCmer (step 1), determination of primary MAGs for isolates (step 2) and for uncultivated components (step 3), assignment of contigs with <90% AF to obtain final MAGs for all components except for FC8-9 (step 4) and separation of FC8-9 by using the Naïve Bayesian Classifier (4-nt motif) are shown. MAGs, metagenome-assembled genomes; AF, alignment fraction; PP, posteriori probability. **Figure S2.** Alignment fraction distribution of aligned scaffolds for each isolate. Dashed line, the alignment fraction threshold of 90% for scaffold assignment with high confidence. **Figure S3.** The distribution of scaffold-level sequencing coverage for all unaligned scaffolds assembled by metaSPAdes. **Figure S4.** The distribution of posteriori probabilities for FC8-9 SCG scaffolds based on FC2 MAG. Only the SCG scaffolds harboring SCGs with >1 copiesare shown here. Posteriori probability was calculated by using the Naïve Bayesian Classifier (4-nt motif). **Figure S5.** Reconstruction statistics for isolates. A and B, for FC2 in terms of scaffold number and base pair (bp) respectively; C and D, for FC3 in terms of scaffold number and bp respectively; E and F, for FC5 in terms of scaffold number and bp respectively; G and H, for FC7 in terms of scaffold number and bp respectively. **Figure S6.** Reconstruction statistics for uncultured components. A and B, for FC1 in terms of scaffold number and base pair (bp) respectively; C and D, for FC4 in terms of scaffold number and bp respectively; E and F, for FC6 interms of scaffold number and bp respectively; G and H, for FC8 in terms of scaffold number and bp respectively; I and J, for FC9 in terms of scaffold number and bp respectively. **Figure S7.** The average amino-acid identity distance between the closest relative and the reference with the second highest SCG AAI for each component. The solid line indicates the distance. For the closest relatives, please refer to Additional file 2: Table S3. **Figure S8.** The heatmap showing the amino-acid identities for SCGs of all components. The reference in red, the closest relative for all components or an additional second closest relative for FC1. For the closest relatives, please refer to Additional file 2: Table S3. **Figure S9.** Statistics of all Firmicutes SCGs. A, percentage of genomes with >1 copies; B, assembly status for all Firmicutes genomes; C, number distribution of SCGs for all Firmicutes genomes. The data were from all 27,565 Firmicutes genomes. Red, SCG with >1 copies in at least one F1RT MAG; dashed line, the median for percentage of genomes with >1 copies at 0.52. **Figure S10.** The sequencing coverage distribution for isolates. A, for FC2; B, for FC3; C, for FC5; D, for FC7. Sequencing coverage is calculated using a fixed window of 500 bp with 250 bp overlap. **Figure S11.** The sequencing coverage distribution for uncultivated components. A, for FC1; B, for FC4; C, for FC6; D, for FC8; E for FC9. Sequencing coverage is calculated using a fixed window of 500 bp with 250 bp overlap. **Figure 12.** Classification statistics of 10-kb fragmentsfor all F1RT MAGs or their reference genomes. A, for F1RT MAGs; B for F1RT reference genomes. The number in a cell is the fraction (%) of fragments classified into their corresponding organism and used as a basis for color intensity. The 10-kb fragments are produced *via *dividing (pre-concatenated) MAGs or reference genomes. For references, please refer to Additional file 2: Table S4. **Figure 13.** Phylogenetic relationships for F1RT MAGs and their reference genomes. A, for F1RT MAGs; B, for reference genomes. Phylogenetic relationships were determined on the basis of the average amino acid identity for SCGs. For references, please refer to Additional file 2: Table S4. **Figure 14.** Mapping qualities for scaffolds with abnormally high or normal sequencing coverage. A, for FC1; B, for FC2; C, for FC3; D, for FC4; E, for FC5; F, for FC6; G, for FC7; H, for FC8; I, for FC9. MAPQ, mapping quality. **Figure 15.** Statistics of duplicated reads mapped to scaffolds with abnormally high or normal sequencing coverage. A, for FC1; B, for FC2; C, for FC3; D, for FC4; E, for FC5; F, for FC6; G, for FC7; H, for FC8; I, for FC9. Duplication is calculated as the total alignments divided by the alignments after removing duplication by using “samtools markdup -r”. **Figure 16.** Size statistics of scaffolds with abnormally high or normal sequencing coverage. A, for FC1; B, for FC2; C, for FC3; D, for FC4; E, for FC5; F, for FC6; G, for FC7; H, for FC8; I, for FC9. **Table S1.** Mapping summary and the read mapping ratios for the references of the two scaffolds.** Table S2.** The genomic statistics of F1RT isolates. **Table S3**. The close relatives of all F1RT components.** Table S4.** Reference genomes of all F1RT components for TETRA analysis.** Table S5.** Alignments between scaffolds with abnormally high sequencing coverage and plasmid sequences.

Additional file 3. Coordinates for nucleotide-based alignments between scaffolds from the FC2 isolate genome and scaffolds from the F1RT metagenome assembled by metaSPAdes.

Additional file 4. Coordinates for nucleotide-based alignments between scaffolds from the FC3 isolate genome and scaffolds from the F1RT metagenome assembled by metaSPAdes.

Additional file 5. Coordinates for nucleotide-based alignments between scaffolds from the FC5 isolate genome and scaffolds from the F1RT metagenome assembled by metaSPAdes.

Additional file 6. Coordinates for nucleotide-based alignments between scaffolds from the FC7 isolate genome and scaffolds from the F1RT metagenome assembled by metaSPAdes.

Additional file 7. Sequencing coverage for scaffolds uniquely assembled by metaSPAdes.

Additional file 8. SCG scaffolds and their sequencing coverage for FC8-9.

Additional file 9. SCG scaffolds, their origins and sequencing coverage. Number in brackets, sequencing coverage across a whole scaffold.

## Data Availability

1. The original data for F1RT including both metagenome and high-quality reads were downloaded from the GigaScience database (http://gigadb.org/dataset/100049). 2. The references for the closest relatives and references for reconstruction validation by TETRA used in this study were downloaded from the NCBI database (ftp://ftp.ncbi.nlm.nih.gov/genomes/) on 20 January 2017. They are given in Additional file 2: Table S3 and Table S4. 3. Four isolate genomes generated in this study were deposited into the NCBI database with the BioProject numbers PRJNA792956, PRJNA794355, PRJNA794362, and PRJNA794369 for FC2, FC3, FC5, and FC7 respectively. 4. Nine MAGs were deposited into the NCBI database with the BioProject number PRJNA794931 for FC1-9. 5. For convenience, 4 isolate genomes and 9 MAGs generated in this study as well as codes for the in-house pipeline to generate MAGs were deposited at https://github.com/Yizhuangzhou/F1RT. 6. For convenience, all mapped reads were deposited at https://github.com/Yizhuangzhou/F1RT.

## Abbreviations

MAG: Metagenome-assembled genome
SCG: Single-copy gene
AAI: Average amino acid identity
SCG AAI: SCG-based average amino acid identity
AF: Alignment fraction
NCBI: National Center for Biotechnology Information
ANI: Average Nucleotide Identity
