# Supplementary material for: Constructing metagenome-assembled genomes for almost all components in a real bacterial consortium for binning benchmarking
Source: BMC Genomics. 2022 Nov 10;23:746. doi: 10.1186/s12864-022-08967-x (PMC9647946; doi:10.1186/s12864-022-08967-x)
Supplement: Supplementary file 2 — Figure S1. A schematic flow chart for untangling MAGs for almost all F1RT components. Steps including genome-wide alignment by NUCmer (step 1), determination of primary MAGs for isolates (step 2) and for uncultivated components (step 3), assignment of contigs with <90% AF to obtain final MAGs for all components except for FC8-9 (step 4) and separation of FC8-9 by using the Naïve Bayesian Classifier (4-nt motif) are shown. MAGs, metagenome-assembled genomes; AF, alignment fraction; PP, posteriori probability. Figure S2. Alignment fraction distribution of aligned scaffolds for each isolate. Dashed line, the alignment fraction threshold of 90% for scaffold assignment with high confidence. Figure S3. The distribution of scaffold-level sequencing coverage for all unaligned scaffolds assembled by metaSPAdes. Figure S4. The distribution of posteriori probabilities for FC8-9 SCG scaffolds based on FC2 MAG. Only the SCG scaffolds harboring SCGs with >1 copiesare shown here. Posteriori probability was calculated by using the Naïve Bayesian Classifier (4-nt motif). Figure S5. Reconstruction statistics for isolates. A and B, for FC2 in terms of scaffold number and base pair (bp) respectively; C and D, for FC3 in terms of scaffold number and bp respectively; E and F, for FC5 in terms of scaffold number and bp respectively; G and H, for FC7 in terms of scaffold number and bp respectively. Figure S6. Reconstruction statistics for uncultured components. A and B, for FC1 in terms of scaffold number and base pair (bp) respectively; C and D, for FC4 in terms of scaffold number and bp respectively; E and F, for FC6 interms of scaffold number and bp respectively; G and H, for FC8 in terms of scaffold number and bp respectively; I and J, for FC9 in terms of scaffold number and bp respectively. Figure S7. The average amino-acid identity distance between the closest relative and the reference with the second highest SCG AAI for each component. The solid line indicates the distance. For the [file 12864_2022_8967_MOESM2_ESM.pdf]

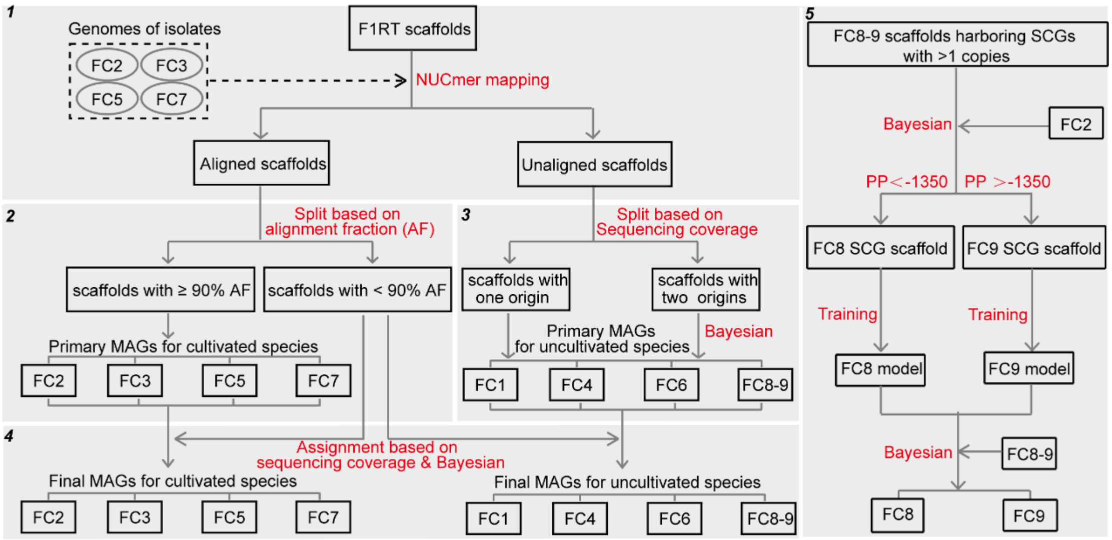

Figure S1. A schematic flow chart for untangling MAGs for almost all F1RT components. Steps including genome-wide alignment by NUCmer (step 1), determination of primary MAGs for isolates (step 2) and for uncultivated components (step 3), assignment of contigs with <90% AF to obtain final MAGs for all components except for FC8-9 (step 4) and separation of FC8-9 by using the Naïve Bayesian Classifier (4-nt motif) are shown. MAGs, metagenome-assembled genomes; AF, alignment fraction; PP, posteriori probability.

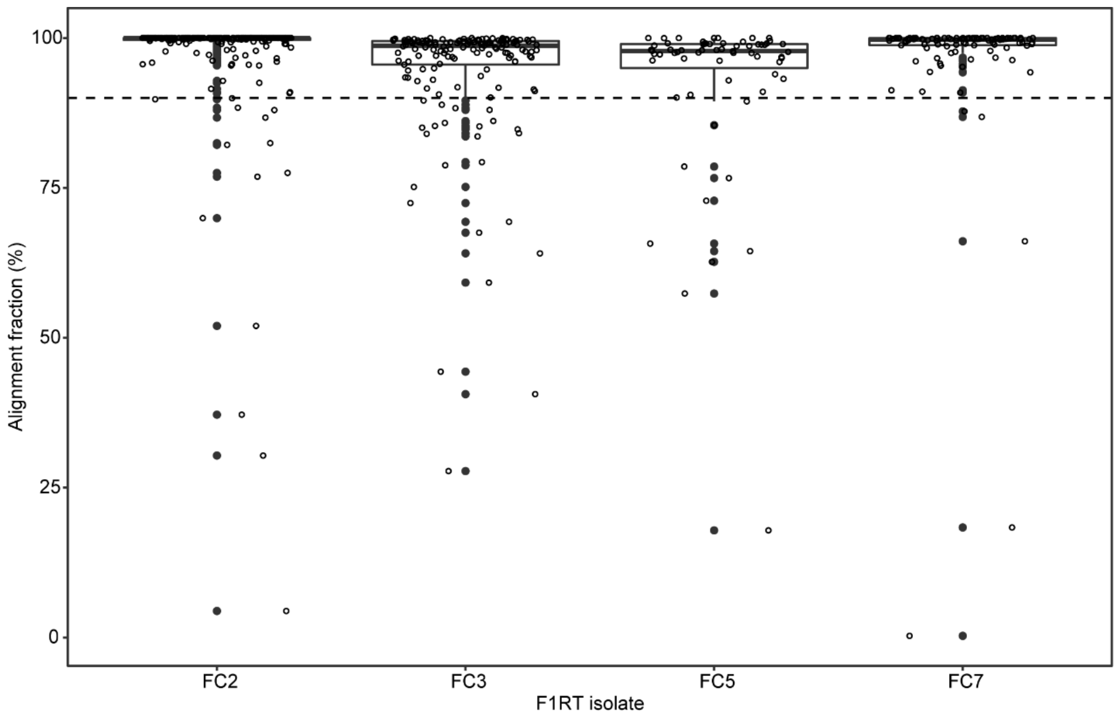

Figure S2. Alignment fraction distribution of aligned scaffolds for each isolate. Dashed line, the alignment fraction threshold of 90% for scaffold assignment with high confidence.

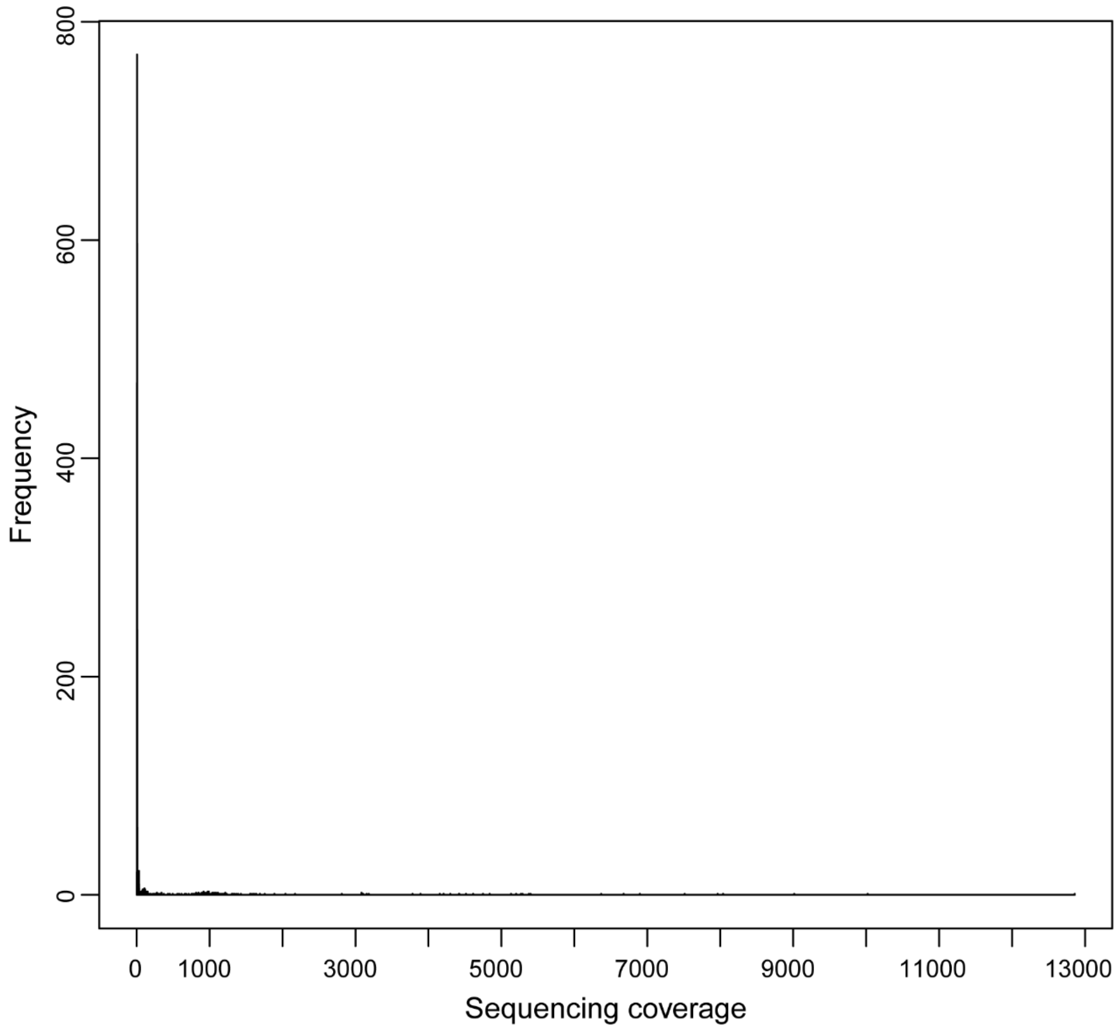

Figure S3. The distribution of scaffold-level sequencing coverage for all unaligned scaffolds assembled by metaSPAdes.

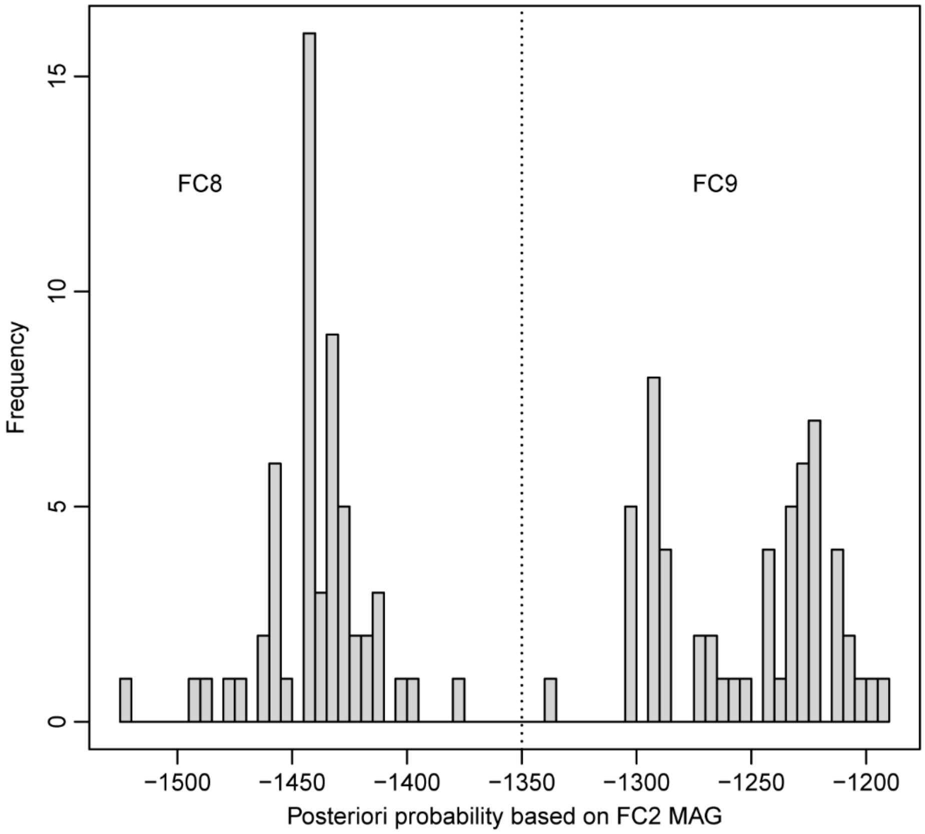

Figure S4. The distribution of posteriori probabilities for FC8-9 SCG scaffolds based on FC2 MAG. Only the SCG scaffolds harboring SCGs with >1 copies are shown here. Posteriori probability was calculated by using the Naïve Bayesian Classifier (4-nt motif).

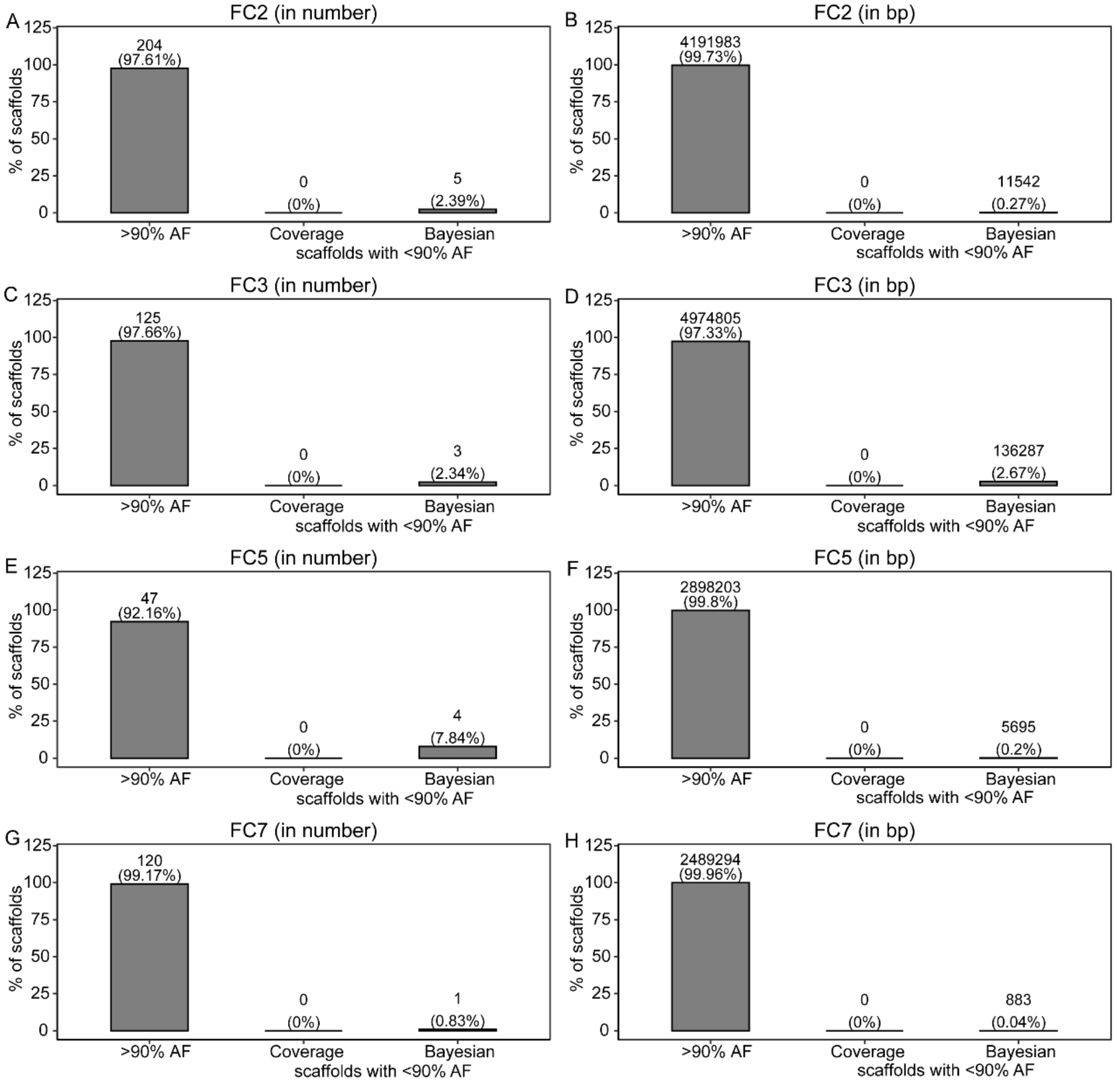

Figure S5. Reconstruction statistics for isolates. A and B, for FC2 in terms of scaffold number and base pair (bp) respectively; C and D, for FC3 in terms of scaffold number and bp respectively; E and F, for FC5 in terms of scaffold number and bp respectively; G and H, for FC7 in terms of scaffold number and bp respectively.

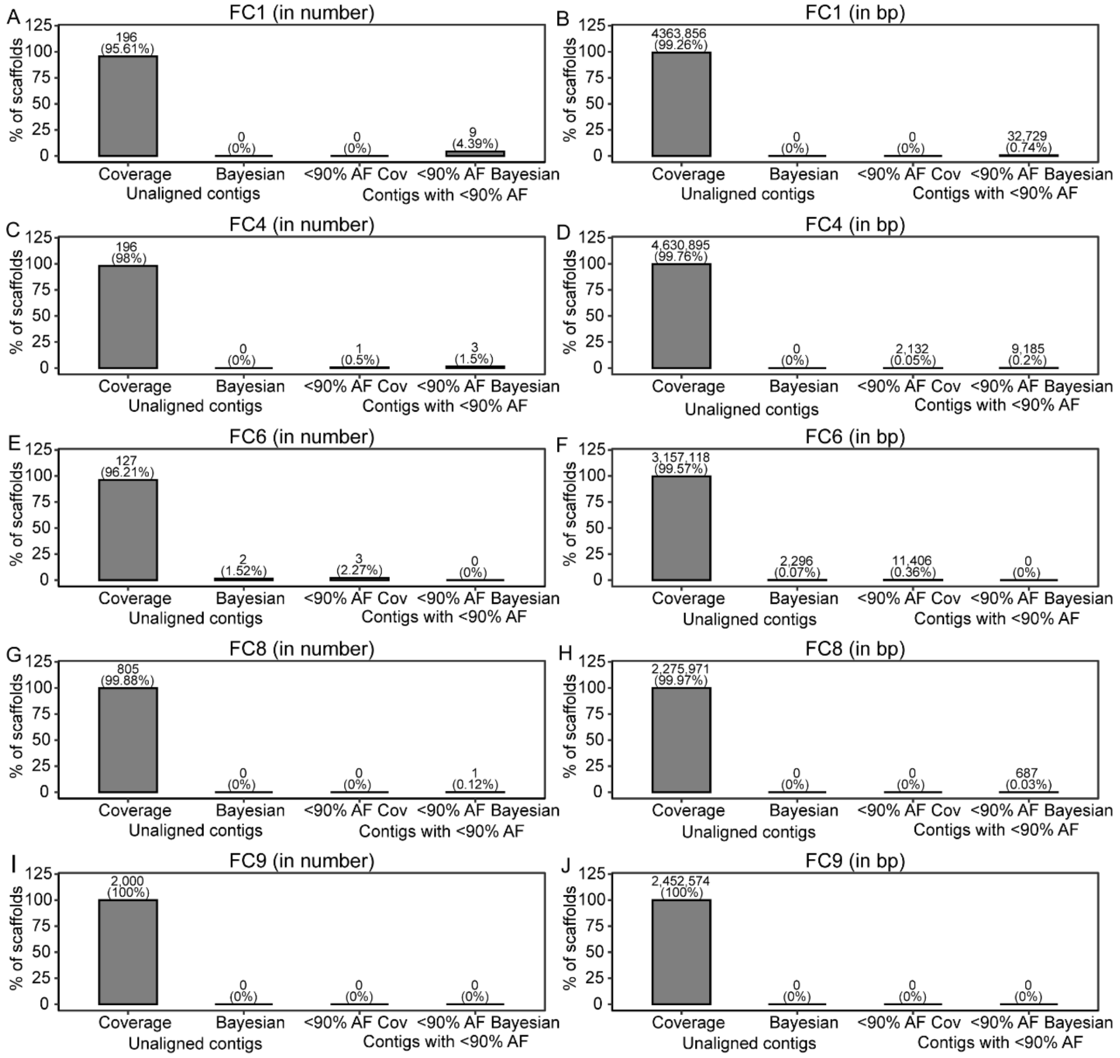

Figure S6. Reconstruction statistics for uncultured components. A and B, for FC1 in terms of scaffold number and base pair (bp) respectively; C and D, for FC4 in terms of scaffold number and bp respectively; E and F, for FC6 in terms of scaffold number and bp respectively; G and H, for FC8 in terms of scaffold number and bp respectively; I and J, for FC9 in terms of scaffold number and bp respectively.

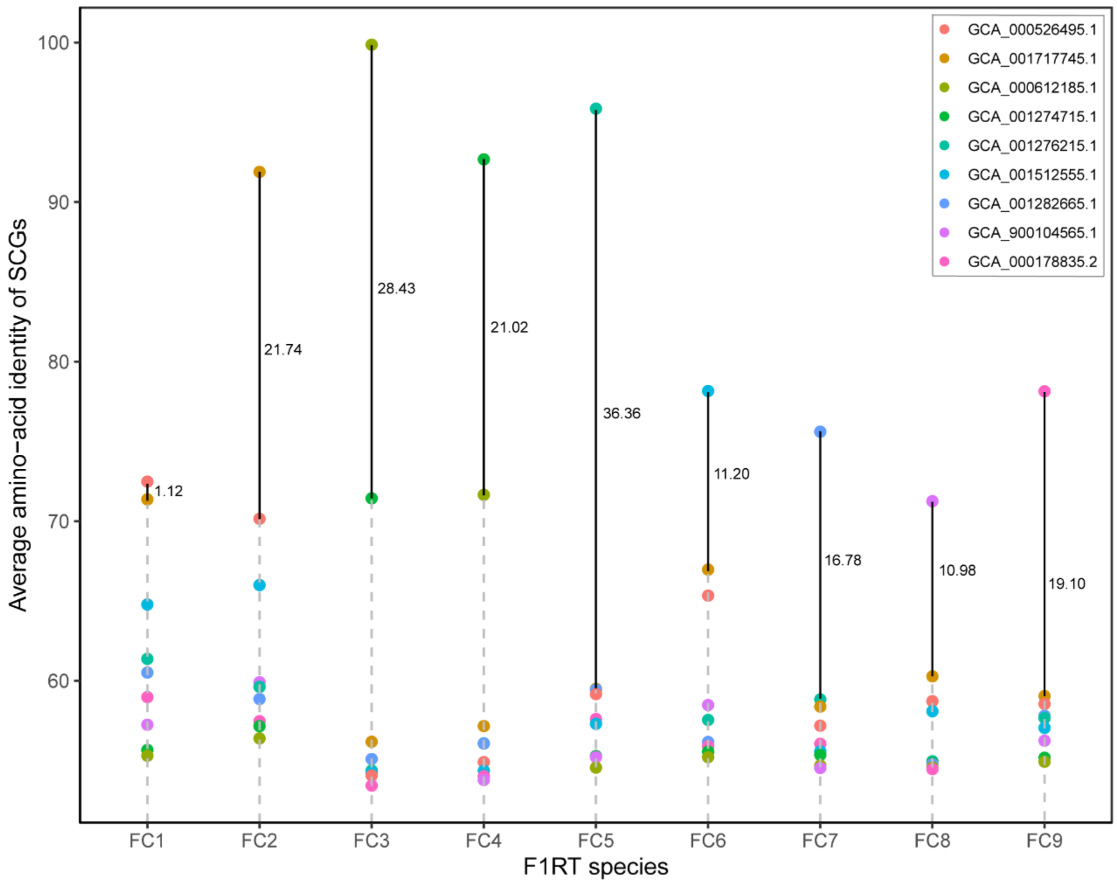

Figure S7. The average amino-acid identity distance between the closest relative and the reference with the second highest SCG AAI for each component. The solid line indicates the distance. For the closest relatives, please refer to Additional file 2: Table S3.

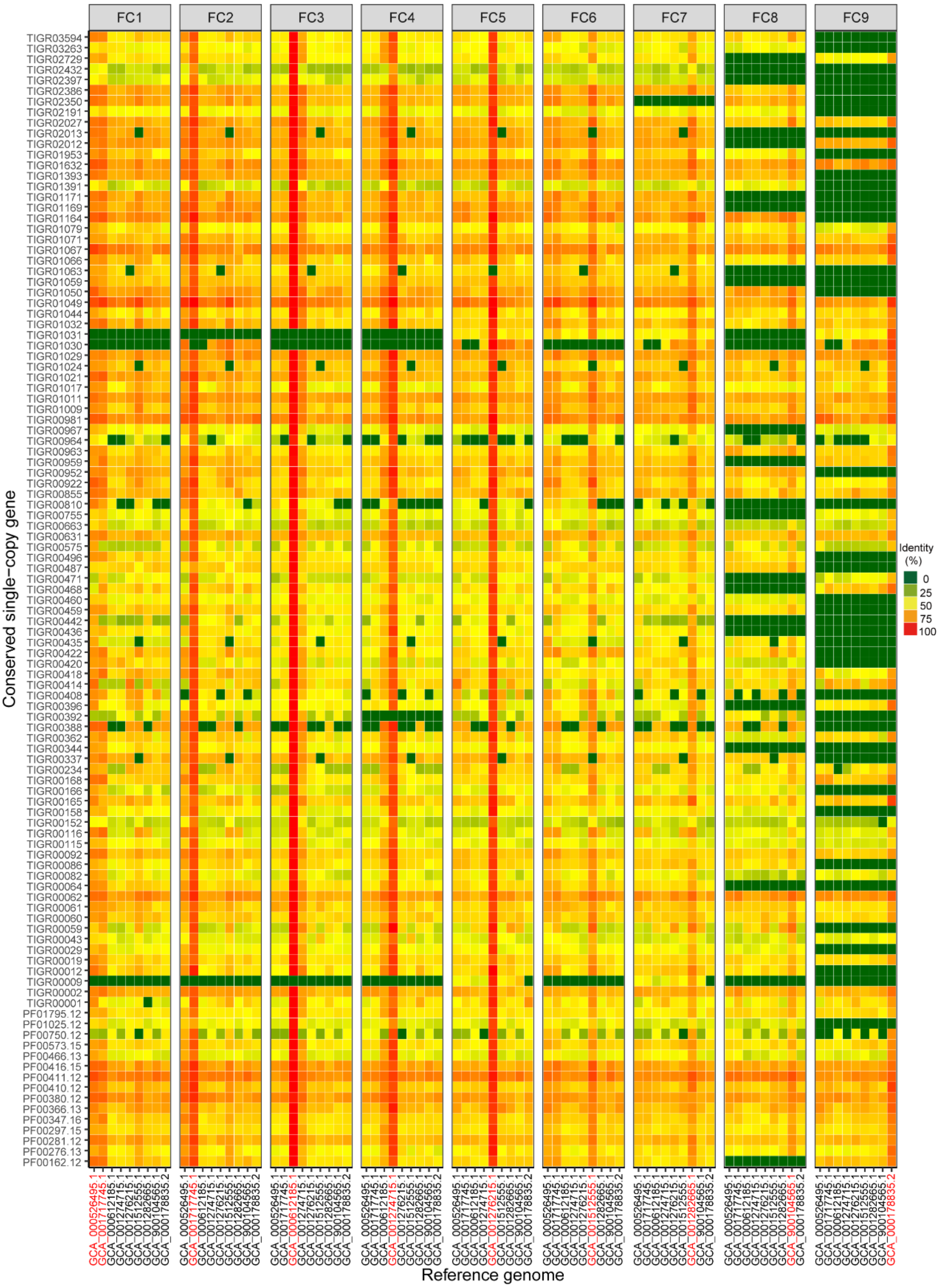

Figure S8. The heatmap showing the amino-acid identities for SCGs of all components. The reference in red, the closest relative for all components or an additional second closest relative for FC1. For the closest relatives, please refer to Additional file 2: Table S3.

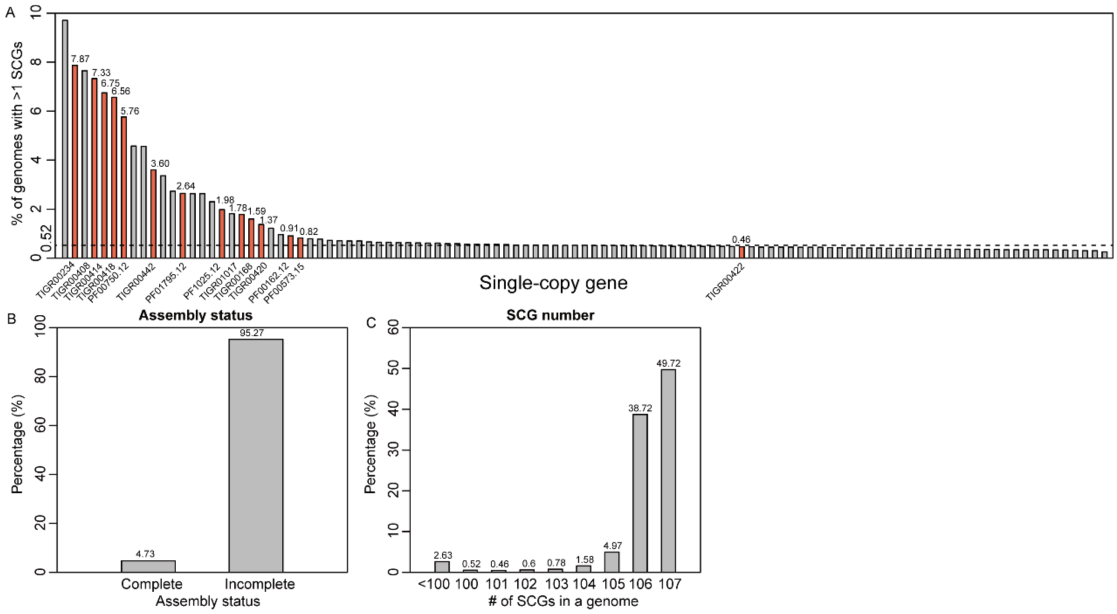

Figure S9. Statistics of all Firmicutes SCGs. A, percentage of genomes with >1 copies; B, assembly status for all Firmicutes genomes; C, number distribution of SCGs for all Firmicutes genomes. The data were from all 27,565 Firmicutes genomes. Red, SCG with >1 copies in at least one F1RT MAG; dashed line, the median for percentage of genomes with >1 copies at 0.52.

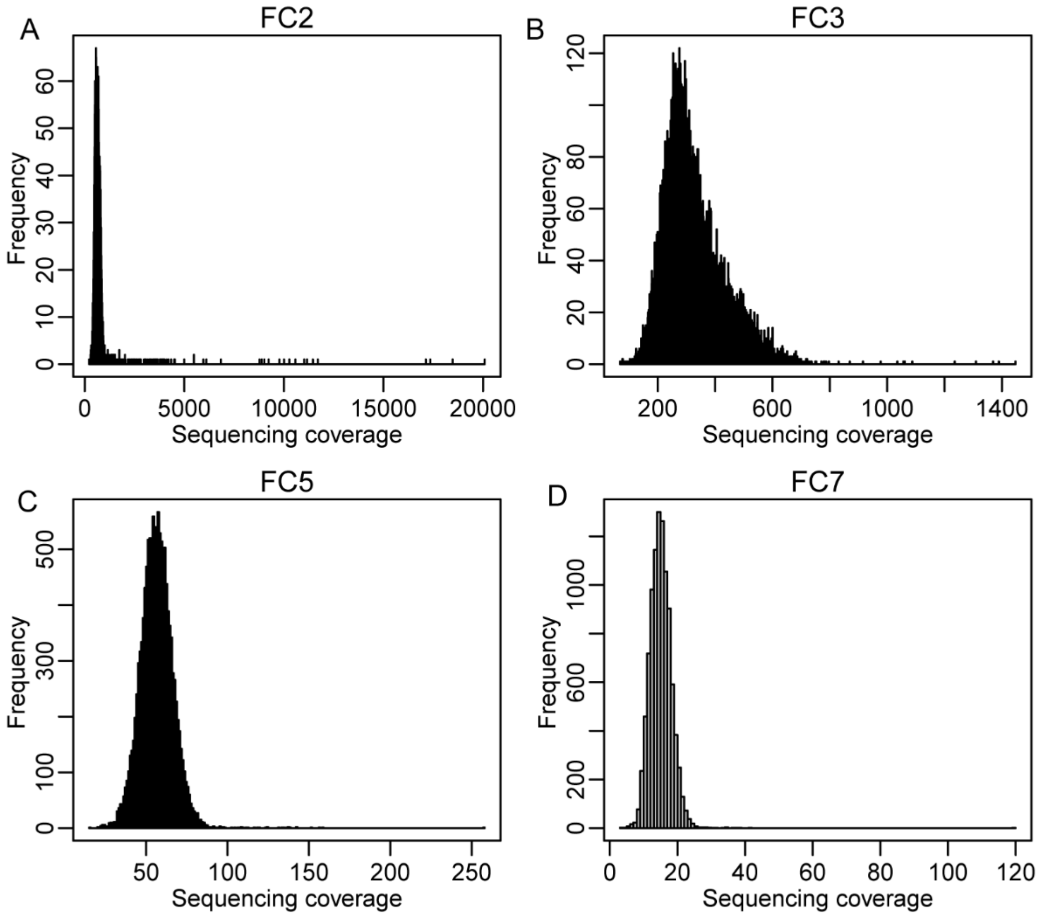

Figure S10. The sequencing coverage distribution for isolates. A, for FC2; B, for FC3; C, for FC5; D, for FC7. Sequencing coverage is calculated using a fixed window of 500 bp with 250 bp overlap.

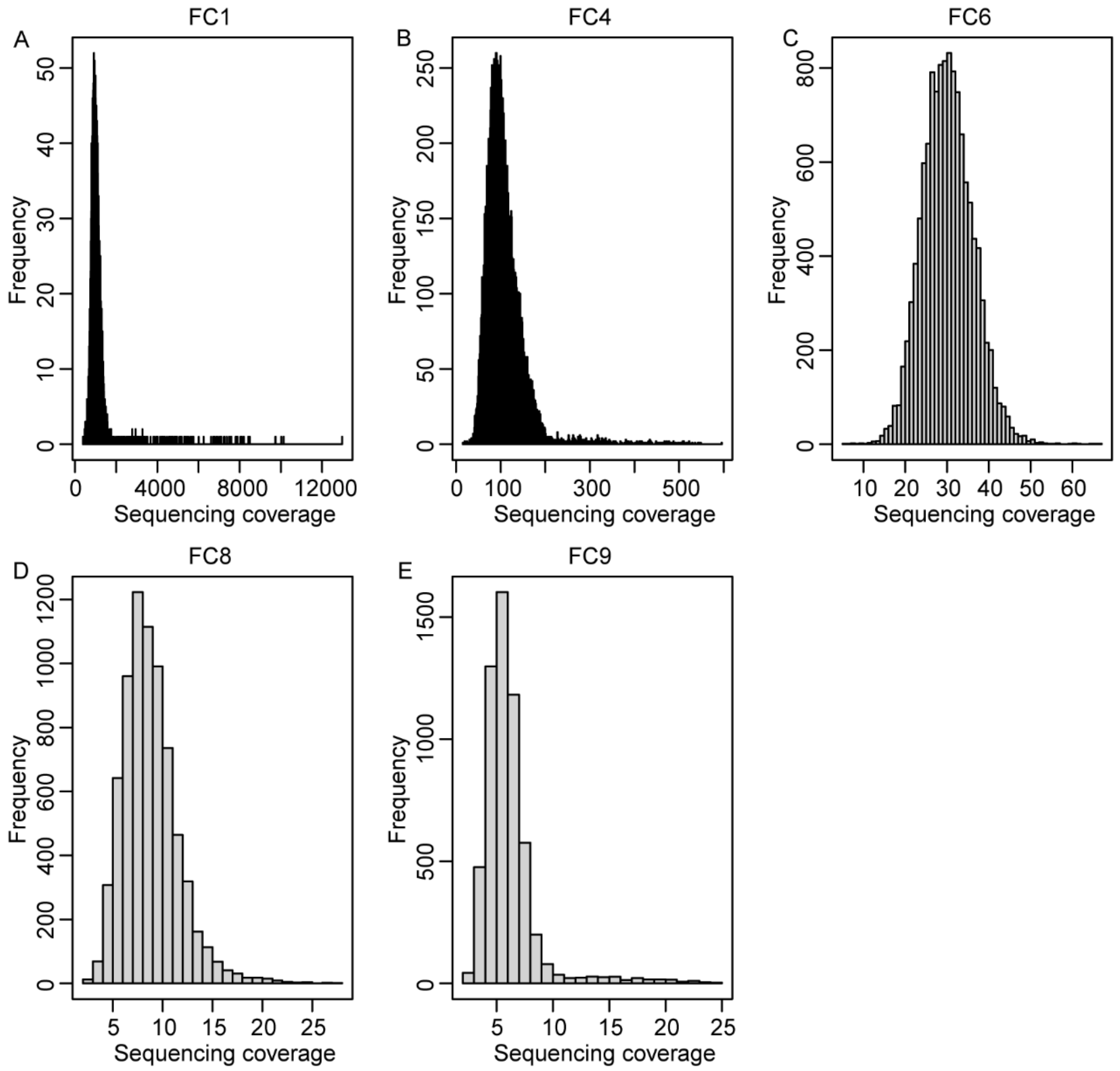

Figure S11. The sequencing coverage distribution for uncultivated components. A, for FC1; B, for FC4; C, for FC6; D, for FC8; E for FC9. Sequencing coverage is calculated using a fixed window of 500 bp with 250 bp overlap.

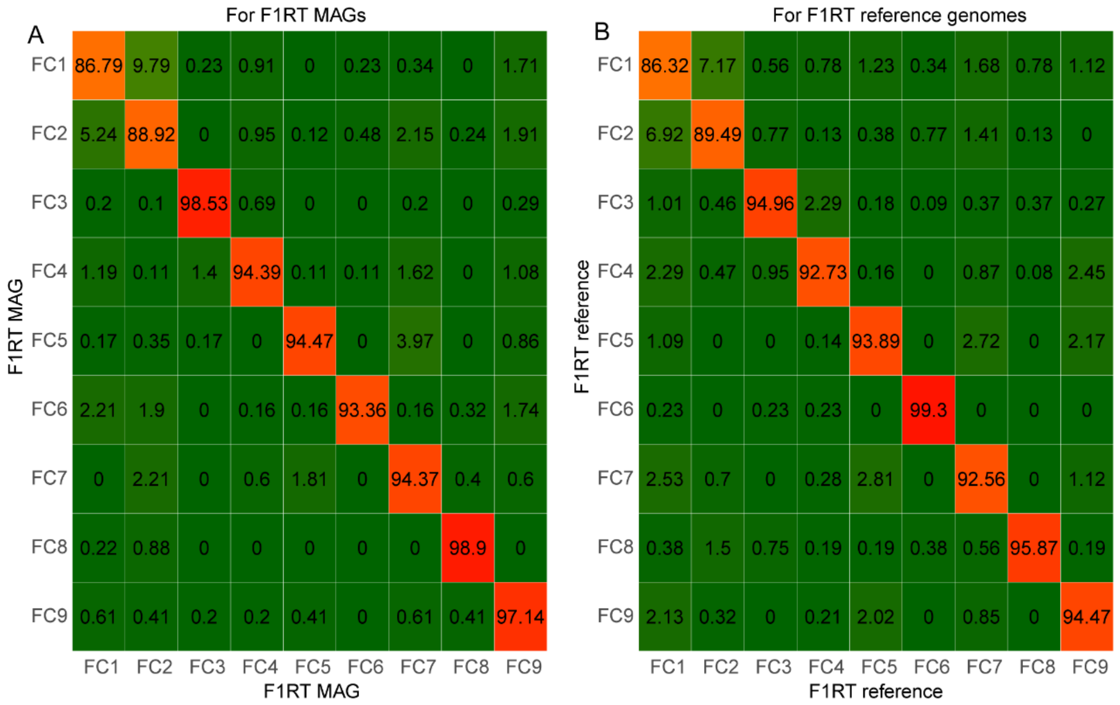

Figure 12. Classification statistics of 10-kb fragments for all F1RT MAGs or their reference genomes. A, for F1RT MAGs; B for F1RT reference genomes. The number in a cell is the fraction (%) of fragments classified into their corresponding organism and used as a basis for color intensity. The 10-kb fragments are produced *via* dividing (pre-concatenated) MAGs or reference genomes. For references, please refer to Additional file 2: Table S4.

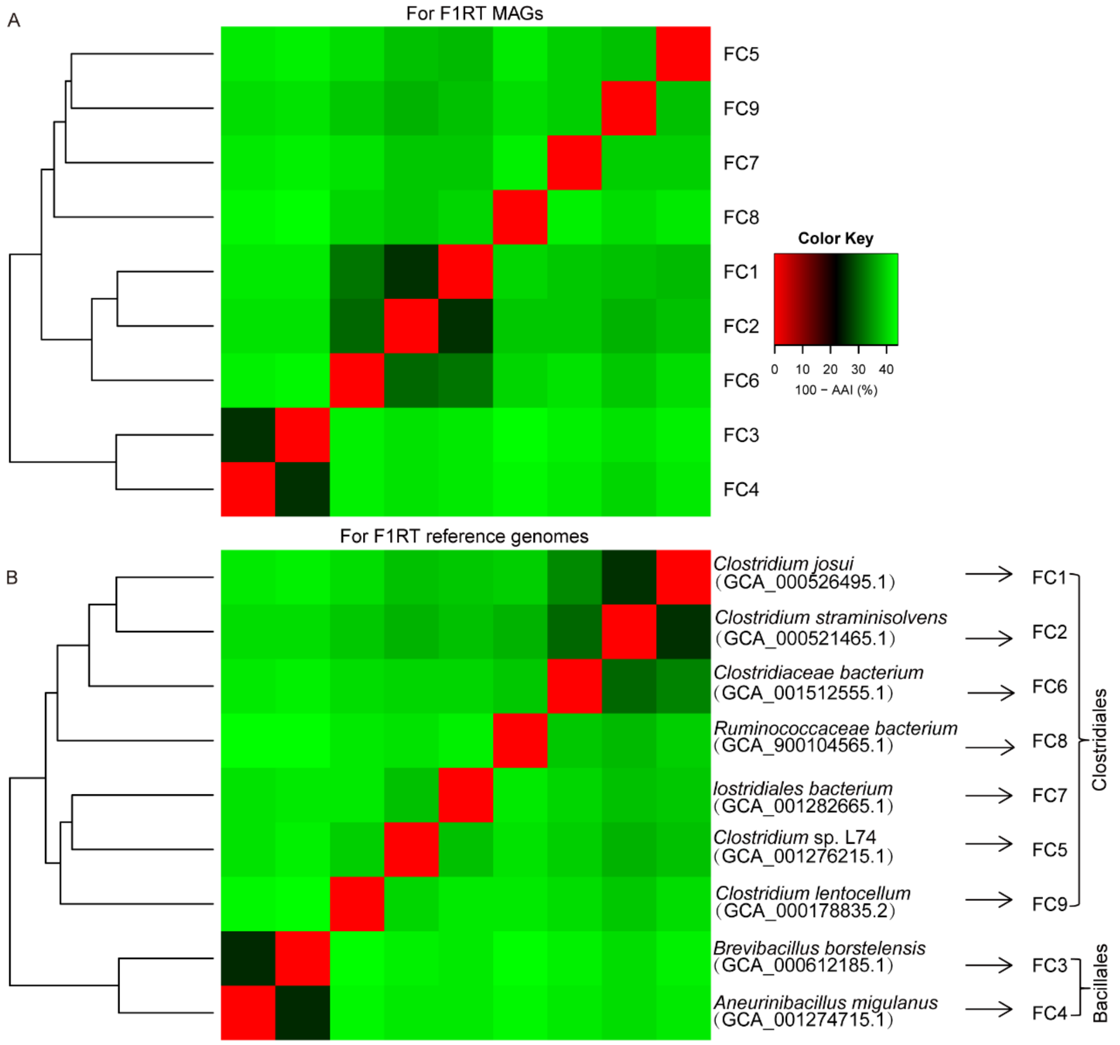

Figure 13. Phylogenetic relationships for F1RT MAGs and their reference genomes. A, for F1RT MAGs; B, for reference genomes. Phylogenetic relationships were determined on the basis of the average amino acid identity for SCGs. For references, please refer to Additional file 2: Table S4.

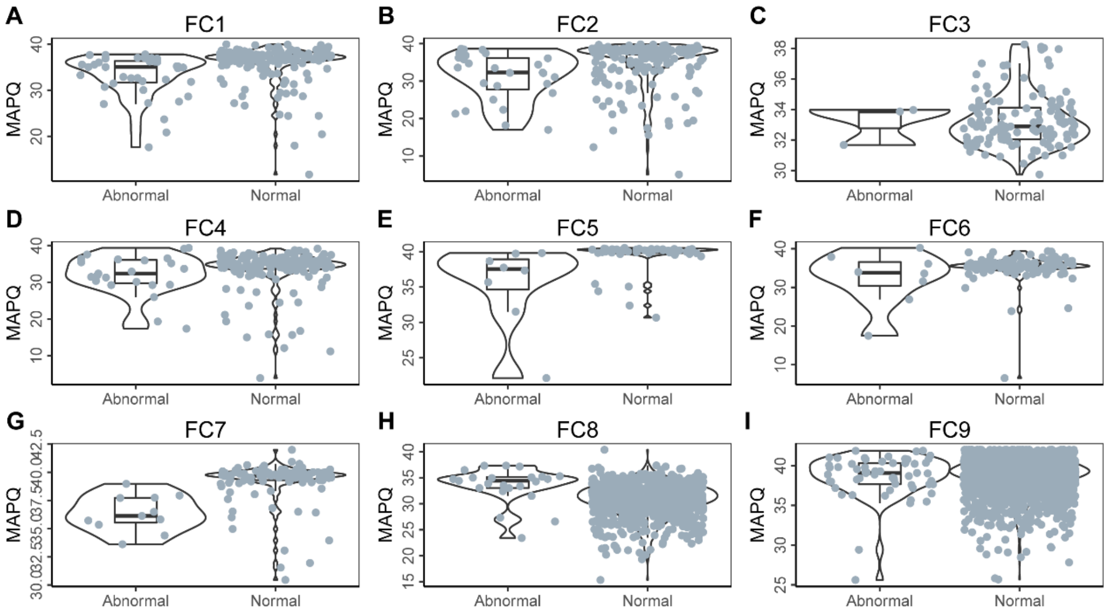

Figure 14. Mapping qualities for scaffolds with abnormally high or normal sequencing coverage. A, for FC1; B, for FC2; C, for FC3; D, for FC4; E, for FC5; F, for FC6; G, for FC7; H, for FC8; I, for FC9. MAPQ, mapping quality.

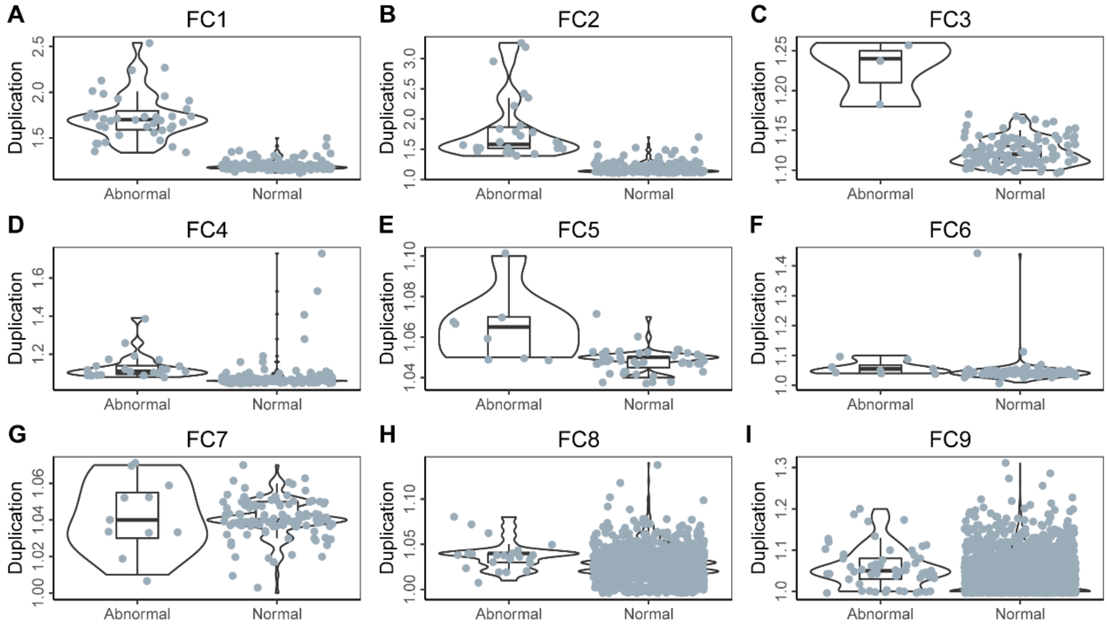

Figure 15. Statistics of duplicated reads mapped to scaffolds with abnormally high or normal sequencing coverage. A, for FC1; B, for FC2; C, for FC3; D, for FC4; E, for FC5; F, for FC6; G, for FC7; H, for FC8; I, for FC9. Duplication is calculated as the total alignments divided by the alignments after removing duplication by using "samtools markdup -r".

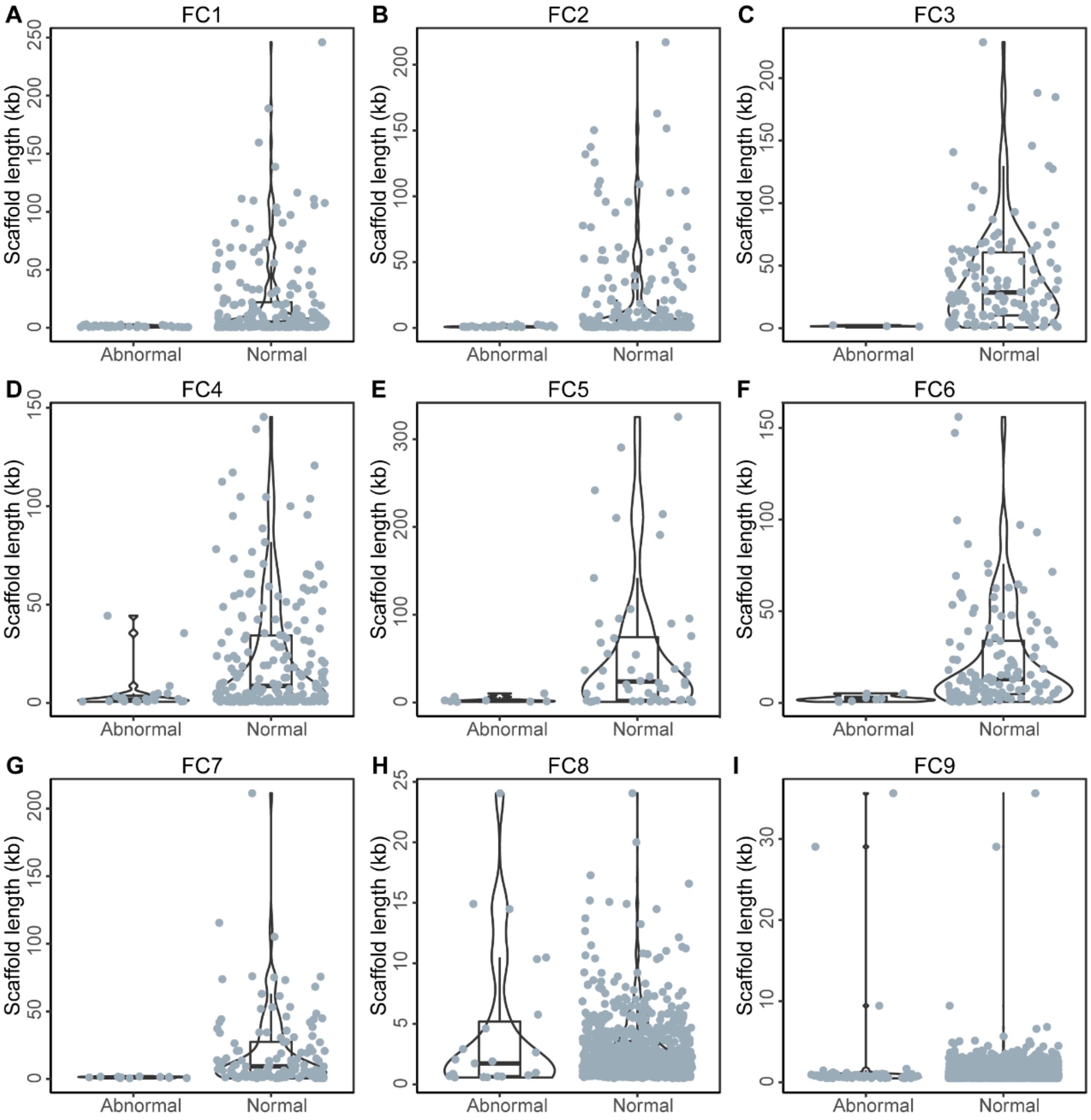

Figure 16. Size statistics of scaffolds with abnormally high or normal sequencing coverage. A, for FC1; B, for FC2; C, for FC3; D, for FC4; E, for FC5; F, for FC6; G, for FC7; H, for FC8; I, for FC9.

Table S1. Mapping summary and the read mapping ratios for the references of the two scaffolds

Scaffold	Len. (bp)	Ide. (%)	Cov. (%)	Reference	# Mapped reads (%)*
NODE_34_length_598_cov_174.909761	598	99.5	100	*Acetivibrio thermocellus* ATCC 27405	16,406 (0.0259%)
NODE_38_length_588_cov_1.273921	588	91.48	99.83	*Heliomicrobium modesticaldum* Ice1	3548 (0.0059%)

Note: *, the mapping ratio (%) is calculated as the number of the initially unmapped reads mapped against the reference independently divided by the total number of reads; Len., length; Ide., Identity; Cov., coverage.

Table S2. The genomic statistics of F1RT isolates.

Attribute	FC2	FC3	FC5	FC7
Sequencing depth	33.18	37.93	36.77	32.02
# of scaffolds	82	123	135	44
Genome size (bp)	4,385,464	5,033,134	2,899,726	2,504,904
Genome net size (bp)	4,381,905	5,010,546	2,859,493	2,500,374
Genome net size (%)	99.92	99.55	98.61	99.82
G+C (%)	38.23	51.88	26.8	32.21
Min length (bp)	517	500	506	610
Mean length (bp)	53,481	40,919	21,479	56,929
N50 (bp)	269,220	73,424	45,460	132,267
N90 (bp)	47,925	25,398	12,861	39,114
Max length (bp)	1,017,469	274,330	245,480	522,162
Assembly completeness (%)[$]	99.93	99.80	99.36	99.91

Note: [$], estimated by mapping reads back to their assembled genomes

Table S3. The close relatives of all F1RT components.

Sp.	Accession	Strain	AAI (%)	Cov. (%)
FC1	GCA_000526495.1	*Clostridium josui* JCM 17888	72.48	99.48
FC2	GCA_001717745.1	*Clostridium* sp. Bc-iso-3	91.89	99.05
FC3	GCA_000612185.1	*Brevibacillus borstelensis* 3096-7	99.86	99.05
FC4	GCA_001274715.1	*Aneurinibacillus migulanus*	92.68	100.00
FC5	GCA_001276215.1	*Clostridium* sp. L74	95.86	98.15
FC6	GCA_001512555.1	*Clostridiaceae bacterium* DTU059	78.16	95.28
FC7	GCA_001282665.1	*Clostridiales bacterium* mt11	75.61	100.00
FC8	GCA_900104565.1	*Ruminococcaceae bacterium* Marseille-P2935	71.26	100.00
FC9	GCA_000178835.2	*Clostridium lentocellum* DSM 5427	78.14	100.00

Note: Sp., species; Cov., coverage of SCGs; AAI, average amino acid identity for SCGs.

Table S4. Reference genomes of all F1RT components for TETRA analysis.

Species	Accession	Strain	From
FC1	GCA_000526495.1	*Clostridium josui* JCM 17888	Table S3
FC2	GCA_000521465.1	*Clostridium straminisolvens* JCM 21531	Table 1
FC3	GCA_000612185.1	*Brevibacillus borstelensis* 3096-7	Tables S3 & 1
FC4	GCA_001274715.1	*Aneurinibacillus migulanus*	Table S3
FC5	GCA_001276215.1	*Clostridium* sp. L74	Table S3
FC6	GCA_001512555.1	*Clostridiaceae bacterium* DTU059	Table S3
FC7	GCA_001282665.1	*Clostridiales bacterium* mt11	Table S3
FC8	GCA_900104565.1	*Ruminococcaceae bacterium* Marseille-P2935	Table S3
FC9	GCA_000178835.2	*Clostridium lentocellum* DSM 5427	Table S3

Table S5. Alignments between scaffolds with abnormally high sequencing coverage and plasmid sequences.

Scaffold	Plasmid	Identity (%)	Match (bp)
NODE_2018_length_1315_cov_2644.201587	CP016544.1	81.54	769
NODE_3347_length_652_cov_1894.559464	CP012645.1	85.37	205
NODE_2074_length_1270_cov_1564.674897	CP010977.1	81.3	524
NODE_2674_length_899_cov_1418.851896	CP010270.1	86.45	641
NODE_3401_length_631_cov_1447.815972	CP003018.1	85.94	631
NODE_3138_length_727_cov_1550.629464	CP011009.1	91.98	720
NODE_3138_length_727_cov_1550.629464	CP003018.1	91.11	727
NODE_2634_length_915_cov_1296.452326	CP010270.1	90.31	287
NODE_2835_length_837_cov_1917.194373	CP010270.1	88.16	768
NODE_3657_length_562_cov_1147.185404	CP010270.1	92.94	489
NODE_3714_length_544_cov_1427.856851	CP011009.1	87.21	542
NODE_2852_length_830_cov_167.801290	CP002282.1	81.54	770
NODE_2852_length_830_cov_167.801290	CP001987.1	87.34	766
NODE_3464_length_613_cov_171.071685	CP016544.1	80.36	430
NODE_3464_length_613_cov_171.071685	CP006375.1	84.16	320
NODE_3172_length_715_cov_43.122727	LNUS01000004.1	81.7	386
